# BMCC1 Is an AP-2 Associated Endosomal Protein in Prostate Cancer Cells

**DOI:** 10.1371/journal.pone.0073880

**Published:** 2013-09-06

**Authors:** Janelle L. Harris, Renée S. Richards, Clement W. K. Chow, Soon Lee, Misook Kim, Marion Buck, Linda Teng, Raymond Clarke, Robert A. Gardiner, Martin F. Lavin

**Affiliations:** 1 Queensland Institute of Medical Research, Herston, Brisbane, Queensland, Australia; 2 University of Queensland Centre for Clinical Research, Herston, Brisbane, Queensland, Australia; 3 School of Medicine, University of Western Sydney, Liverpool, Sydney, Australia; University of Illinois, United States of America

## Abstract

The prostate cancer antigen gene 3 (*PCA3*) is embedded in an intron of a second gene *BMCC1* (Bcl2-/adenovirus E1B nineteen kDa-interacting protein 2 (BNIP-2) and Cdc42GAP homology BCH motif-containing molecule at the carboxyl terminal region 1) which is also upregulated in prostate cancer. BMCC1 was initially annotated as two genes (*C9orf65/PRUNE* and *BNIPXL*) on either side of PCA3 but our data suggest that it represents a single gene coding for a high molecular weight protein. Here we demonstrate for the first time the expression of a >300 kDa BMCC1 protein (BMCC1-1) in prostate cancer and melanoma cell lines. This protein was found exclusively in the microsomal fraction and localised to cytoplasmic vesicles. We also observed expression of BMCC1 protein in prostate cancer sections using immunohistology. GST pull down, immunoprecipitation and mass spectrometry protein interaction studies identified multiple members of the Adaptor Related Complex 2 (AP-2) as BMCC1 interactors. Consistent with a role for BMCC1 as an AP-2 interacting endosomal protein, BMCC1 co-localised with β-adaptin at the perinuclear region of the cell. BMCC1 also showed partial co-localisation with the early endosome small GTP-ase Rab-5 as well as strong co-localisation with internalised pulse-chase labelled transferrin (Tf), providing evidence that BMCC1 is localised to functional endocytic vesicles. BMCC1 knockdown did not affect Tf uptake and AP-2 knockdown did not disperse BMCC1 vesicular distribution, excluding an essential role for BMCC1 in canonical AP-2 mediated endocytic uptake. Instead, we posit a novel role for BMCC1 in post-endocytic trafficking. This study provides fundamental characterisation of the BMCC1 complex in prostate cancer cells and for the first time implicates it in vesicle trafficking.

## Introduction

Prostate cancer is a commonly diagnosed malignancy and a leading cause of cancer deaths worldwide. This condition exhibits a wide spectrum of histological changes and behaviours, ranging from premalignant lesions, to localized prostate cancers that follow an indolent course to a percentage of cancers that metastasize early and progress rapidly [1]. Therefore, in addition to development of improved diagnostic and prognostic tools, a detailed understanding of the cell biology of prostate cancer development and progression is required. One of the most promising biomarkers for prostate cancer is the non-coding RNA PCA3 [2,3]. This RNA was identified as a prostate cancer biomarker by differential display analysis of RNAs from histologically normal and malignant tissue from the same patients, and was originally described as Differential Display clone 3 (DD3) [2]. High levels of PCA3 expression are strongly associated with malignant transformation of the prostate epithelium, however, the biological basis for this correlation has not been elucidated [2–4].

We previously demonstrated that *PCA3* is embedded in the intron of another gene, *BMCC1* [5]. *BMCC1* was originally annotated as two discrete genes *c9orf65/PRUNE* (coding for N-terminal region) and *BNIPXL* (coding for C-terminal region). A *BNIPXL* (*BMCC1-4*) open reading frame was identified by database searching for homologues to the Bcl-2 interacting protein BNIP-2 [6]. The authors identified *BNIPXL/BMCC1-4* as a gene with a region of high *BNIP-2* homology, and named the homologous region the BCH (BNIP-2/Cdc42GAP Homology) domain [6]. Using exogenously expressed tagged BNIPXL proteins, they demonstrated that it interacts with RhoA and its activator proto-Lbc [6]. Coexpression and interaction studies demonstrated that BNIPXL binds RhoA and proto-Lbc via different regions and that BNIPXL expression blocks RhoA signalling, at least partially by inhibiting the stimulatory RhoA-Lbc interaction [6]. Machida et al. [7] identified a longer isoform of BMCC1 in human neuroblastoma (BMCC1-3), which spanned coding exons 7-19 of the later identified BMCC1-1. High levels of expression of this transcript were correlated with a more favourable prognosis for this cancer. Evidence for the expression of the full length BMCC1-1 transcript spanning the *c9orf65* and *BNIPXL* genes was provided by Iwama et al. [8], who cloned a full-length cDNA from Hela cells. In this study and subsequently, BMCC1 transcripts have been detected in restricted regions of the mouse brain [8,9]. Clarke et al. [5] demonstrated that BMCC1 RNA expression is elevated in prostate cancer and metastases compared with benign tissue, indicating that BMCC1 may be functioning differently in different cancers and tissue types. An overview of BMCC1 isoform expression and the diverse functions of BCH domain containing proteins is provided in recent publications [10,11].

Initial identification of a protein corresponding to BMCC1-1 was demonstrated by Iwama et al. [8]. This group raised an antibody specific to the extreme N-terminal region of BMCC1-1, and although multiple bands were detected by Western blot of Hela MR, HEK293T and KNS81 glioma cells, only bands >250 kDa were sensitive to specific siRNA depletion. Mass spectrometry was used to identify these high molecular weight bands as BMCC1-1. In the same study, expression of exogenous protein from a cDNA clone also produced multiple bands on SDS-PAGE, probably indicating substantial proteolysis, making interpretation difficult. More recently, Li et al. [12] identified olfaxin proteins corresponding to alternative BMCC1 transcription start sites expressed in the olfactory bulb, with the predominant band at 52 kDa. Arama et al. [11] detected a band of the same size in whole brain lysates and cultured neurons and astrocytes, which they called BMCC1s. In the present study, expression and initial characterisation of a single 340 kDa BMCC1 protein in the prostate cancer cell line LNCaP is described. The identity of this protein was robustly established by detection with several antibodies to C- and N-terminal regions of the protein, depletion by specific siRNA and supported by MALDI TOF/TOF analyses. For the first time we identified endogenous protein interactions involving a region outside the BCH domain. We found that a BMCC1 region without computationally identifiable functional domains interacts with the Adaptor protein complex 2 (AP-2). We also demonstrated co-localisation of BMCC1 with β-adaptin and various endosomal markers, including Rab5 and internalised transferrin (Tf). Preliminary functional analyses suggest BMCC1 is a non-canonical AP-2 interacting protein involved in post-endocytic trafficking.

## Materials and Methods

### Tissues and cell lines

Human prostate tissues were donated by patients at the Royal Brisbane Hospital with written informed consent under ethical approval of the Queensland Institute for Medical Research human ethics committee. Patient consent forms have been retained. Mouse tissues were obtained from a5 month old wild-type male BalbC mouse, under ethical approval of the Queensland Institute for Medical Research (QIMR) animal ethics committee. LNCaP, 22Rv1, DU145, RWPE1, ALVA, PC3 and MCF-7 were obtained from the ATCC. A11, D11, D28, D33 and D38 were gifts from Chris Schmidts laboratory (QIMR). These were obtained from patients enrolled in clinical trials at QIMR, with written informed consent from the QIMR human research ethics committee. A microarray study on the cell lines A11 and D11 has been published [13], as has a clinical study on the D-series patients [14]. All cell lines were maintained in DMEM (Gibco) with penicillin and streptomycin, supplemented with 10% heat inactivated foetal bovine serum (Gibco).

### RNA Extraction and cDNA Synthesis

Total RNA from cell lines and tissues was purified using Trizol (Invitrogen) according to the manufacturers’ instructions. This RNA was reverse transcribed into cDNA using Superscript III (Invitrogen) according to the manufacturers’ instructions.

### PCR and Generation of cDNA clones

cDNA clones of BMCC1 and other genes were generated by amplification of the target from LNCaP cDNA. Oligonucleotides were purchased from Sigma Aldrich, standard PCR amplifications were performed wth *Pfu* Turbo (Roche). Typical cycle conditions were 92°C 2 minutes followed by; 92°C for 30 sec, 60°C for 30 sec, 72°C for 90 sec/kb (2 cycles); 92°C for 30 sec, 57°C for 30 sec, 72°C for 90 sec/kb (2 cycles); 92°C for 30 sec, 53°C for 30 sec, 72°C for 90 sec/kb (30 cycles). All q-PCR was performed using the Corbett Rotorgene-6000 (Qiagen, Australia), Rotorgene-6000 Series Software (Qiagen, Australia) and Quantitect® SYBR® Green PCR Kit (Cat. No. 204143, Qiagen, Australia). Reactions were prepared in triplicate and each contained 7.5µl of qPCR master mix, 0.5µl of each 10µM forward and reverse primer and 5µl of diluted cDNA (1:10 dilution). Cycling condition for BMCC1 and β_2_M primers were as follows: 95°C for 15 min, followed by 45 cycles of 95°C for 20 sec, 58°C for 20 sec and 72°C for 20 sec. Cycling conditions for AP2M1 primers were as follows: 95°C for 15 min, followed by 45 cycles of 95°C for 20 sec, 60°C for 20 sec and 72°C for 20 sec. Data were generated with the Rotorgene-6000 Series Software and relative gene expression levels were calculated using methodology described in Pfaffl [15]. Primer sequences and restriction sites are indicated in supplementary material (Table S1). All restriction enzymes were purchased from NEB.

### Recombinant protein expression, purification and crosslinking

Recombinant protein expression and purification and crosslinking were performed as previously described [16].

### Antibodies

Recombinant BMCC1-GST fusion proteins were expressed and purified as described above. The rabbit antibodies Ab-1 and Ab-3 were generated by injection of rabbits with eluted, non-denatured GST fusion proteins (using the Institute for Medical and Veterinary Science, Adelaide standard dose rate, schedule and adjuvants). BMCC1 Ab-2 antiserum was generated by inoculation of a rabbit with denatured GST fusion protein embedded in unfixed polyacrylamide gel slices (IMVS). BMCC1 sheep antiserum was generated by inoculation of a sheep with eluted non-denatured BMCC1-GST fusion protein (IMVS) (Ab-4). All proteins had N-terminal GST tags when inoculated and the specific BMCC1 sequences for each antibody are detailed in Table S1. Specific antibodies and non-specific control antibodies were purified from serum as described [16].

Commercial antibodies used in this project were mouse anti-DNA PKcs (Santa Cruz product 18-2), mouse anti- RNA polymerase II (Abcam, ab5408), rabbit anti-β-adaptin (which detects AP-1B1 and AP-2B1) (Santa Cruz A-5), mouse anti-EEA1 (BD Transduction Laboratories, 610457), mouse anti-α-tubulin (Sigma T9026) and mouse anti-gapdh (Millipore MAB374). Appropriate Alexafluor conjugated donkey secondary antibodies were obtained from Invitrogen and HRP conjugated secondary antibodies were from Sigma.

### Plasmid and siRNA transfection

LNCaP were plated into 6 well dishes in antibiotic free growth medium at least 48 h before transfection. Plasmids were transfected into LNCaP using Lipofectamine 2000 (Invitrogen) according to the manufacturer’s instructions (2 µg of plasmid and 5 µL of Lipofectamine 2000 per 9.5 cm^2^ well). siRNA duplexes were purchased from Invitrogen (sequences in Table S1) and transfected using Lipofectamine RNAiMAX or Lipofectamine 2000 (100 pmol of siRNA duplex and 5 µL of Lipofectamine per 9.5 cm^2^ well).

### Generation of cell and tissue lysates

Cells were detached from their growth substrate by trypsinization in serum free DMEM with 0.025% trypsin (Gibco), and trypsin was inactivated by addition of DMEM containing 10% serum. Cells were pelleted by centrifugation (500 x g for 5 min) and washed twice in PBS. Washed cell pellets were then lysed in ice-cold Mammalian Cell Lysis Buffer (MCLB, 50 mM Tris pH 7.4, 150 mM NaCl, 1 mM EDTA, 1% NP-40). All lysates contain a 2 x concentration of Roche Complete EDTA-free protease inhibitor. Thorough washing to remove cell culture trypsin and the addition of a high concentration of protease inhibitors are *critical* to detect undegraded BMCC1, which is otherwise subject to rapid proteolysis. Tissue lysates were generated by homogenization in MCLB, by grinding in a Kontes size 22 ground-glass dounce homogenizer. Cell and tissue lysates were cleared by centrifugation (17,000 x g for 20 min at 4°C). Brain tissue lysates were subjected to additional processing to remove a layer of floating lipids. Brain tissue lysates were separated on to Biorad Micro Biospin desalting chromatography columns which had been pre-equilibrated with MCLB, which removed sticky insoluble material. Protein concentrations were determined using the Biorad, Lowry protein assay with BSA standards.

### Western blotting

Samples in Laemelli SDS-PAGE loading buffer were subjected to standard SDS-PAGE. Proteins were transferred onto Amersham nitrocellulose in transfer buffer (6 g/L tris base, 3 g/L glycine, 0.36 g/L SDS, 20% methanol) for 100 V.h. Membranes were blocked in blocking solution (PBS-0.05% Triton-X100 with 5% BSA or PBS/0.07% Tween 20 with 4% skim milk powder). Membranes were probed using between 0.2 and 5 ng/µL of the indicated antibodies in blocking solution at ambient temperature for 2 h or 4°C overnight and detected using HRP conjugated secondary antibodies and Western Lightning luminol reagent (Pierce).

### Immunoprecipitation and GST pulldowns

Cell lysates were generated as described above. For immunoprecipitation, lysates were incubated with purified null or BMCC1 antibodies (0.5-2 µg of antibody per mg of lysate) at 4°C overnight. BMCC1-antibody complexes were precipitated by addition of protein G agarose resin for 2 h at 4°C. For GST pulldowns, lysates were incubated with BMCC1-GST fusion protein crosslinked resin at 4°C overnight. In both cases, unbound proteins were washed from the resin in 3 x 20 bed volume washes of cold lysis buffer. Precipitated proteins were eluted by addition of 2 resin bed volumes of SDS-PAGE loading buffer. Eluted proteins were then analysed by SDS-PAGE and western blotting.

### Mass Spectrometry

Silver stained gel pieces were destained in 15 mM potassium ferricyanide and 50 mM sodium thiosulfate and washed in water. Pieces were dehydrated in 25 mM ammonium bicarbonate/ 50% acetonitrile and vacuum dried. They were sequentially reduced and alkylated with 25mM DTT and 55mM iodoacetamide, both in 25mM ammonium bicarbonate. Gel pieces were dehydrated in 25 mM ammonium bicarbonate / 50% acetonitrile and dried again. They were then rehydrated with 20ng/µL sequencing grade trypsin (Promega) in 40mM ammonium bicarbonate/ 10% acetonitrile, topped up to prevent drying and digested overnight at 37^°^C. Peptides were extracted by washing the pieces in 0.1% TFA. Eluted peptides were desalted using C-18 ZipTips (Millipore) according to the manufacturers instructions. Peptides were then mixed with MALDI matrix (Bruker) and analysed using Bruker Ultraflex in positive ion reflector mode with a MS/MS tolerance of 100 ppm. Analysis was performed in-house using Mascot, searching against the 2012 mammalian non-redundant database, allowing for default post-translational modifications and up to one missed trypsin cleavage [17].

### Purification of microsomes

LNCaP cells were separated into nuclear and cytoplasmic fractions by douncing in hypotonic solution as previously described [16]. Microsomes were purified from cytoplasmic extract by ultracentrifugation (100,000 x g for 1 h at 4°C using a Beckman SW55Ti rotor). Microsomes were resuspended in SDS lysis buffer (2% SDS, 1mM DTT, 125mM Tris pH 6.8) and cleared at 17,000 x g for 20 min. Cytosol was concentrated to 1/10 its original volume using a 10K cut-off centrifugal filter unit (Millipore) before SDS-PAGE.

### Immunohistological staining

A standard immunohistochemistry staining procedure was followed. Paraffin-embedded sections were cut at 4 µm and were mounted on 5-aminopropyltriethoxysilane (AAS) coated slides, which were then left to dry overnight. The paraffin-embedded sections were dewaxed in xylene and were treated with microwave heating at 60°C for 20 min in a citrate buffer (2.1g/1000 ml; pH 6.0) for antigen retrieval. After blocking of endogenous peroxidase, washing in phosphate-buffered saline (PBS) and blocking of non-specific binding of secondary antibody with normal swine serum, routine streptavidin-biotin-peroxidase immunostaining with diaminobenzidine was applied. The sections were incubated overnight in BMCC1 Ab-3. The primary antibody was substituted with PBS in sections used as negative controls. The sections were counterstained with Harris Haematoxylin. Since BMCC1 is known to be positive in benign prostatic tissues, a core of prostate tissue with benign hyperplasia was included as a positive control in each of the array.

Scoring of the BMCC1 protein expression within the cells of the tissues of the 74 patients studied, was based on the proportion of cells stained with the BMCC1 polyclonal antibody and the intensity of the stain within the cells. Staining intensity was scored on the following scale: No staining, mild, moderate and strong. All the samples were analysed under microscopic examination using an Olympus microscope (Model: BX40), at a magnification of x200.

### Immunofluorescence and microscopy

Cells were seeded onto sterile coverslips at least 24 h before fixation. Media was aspirated and cells were washed once in PBS before crosslinking (in PBS with 10% foramlin for 10 min at room temperature). The fixative was removed and cells washed in PBS before permeabilization and blocking in PBS with 0.05% Triton-X100 and 5% FBS. Cells were then stained with between 0.4 and 2 ng/µL of the indicated primary antibodies at ambient temperature for 2 h or 4°C overnight. Species matched purified IgG was employed as an appropriate control for antibody crossreactivity/adsorption. All antibodies were detected using appropriate Alexafluor488 or Alexafluor 594 donkey secondary antibodies. Confocal microscopy was carried out on a Zeiss LSM710. Co-localisation was quantified using Zen 2011 software. Overlap coefficient/overlap coefficient after Manders, a method insensitive to differences in signal intensity, was used to quantify co-localisation in image pairs.

### Transferrin uptake

LNCaP were seeded onto sterile coverslips 24h before the labelling. Pulse-chase uptake of a conjugated transferrin-receptor complex (Tf-R) was performed essentially as described by Aschenbrenner et al. [18]. Briefly, cells were serum starved in serum free media (phenol-red free DMEM-F12, Life Technologies) for 2 h at 37°C. To surface label cells with conjugated transferrin, cells were placed on ice for 30 min then incubated with 20µg/ml Alexafluor-594 conjugated transferrin (Molecular Probes) diluted in ice-cold serum free media for 30 min. Cells were washed twice with ice-cold serum free media to remove unbound transferrin and t=0 coverslips were harvested for BMCC1 immunofluorescence, as described. The remaining coverslips were chased in pre-warmed serum free media for the indicated time periods before harvest. Uptake of conjugated transferrin-receptor complex was also performed using a method similar to Boucrot et al. [19]. Cells were rinsed once with DMEM-F12/5%FCS then incubated for 10 min with 10µM Alexafluor-594 conjugated transferrin in DMEM-F12/5% FCS. Cells were then washed with ice cold PBS before being harvested for immunofluorescence.

## Results

### BMCC1-1 protein is expressed in select cancers

*hBMCC1* is a large and complex transcriptional unit, with the routinely used prostate cancer biomarker *hPCA3* embedded in *BMCC1* intron 6. *BMCC1* has been previously annotated as discrete *PRUNE2* and *BNIP* genes flanking *PCA3*, with exogenous expression based studies reflecting this. Recent results have shown that *BMCC1* produces a transcript (denoted BMCC1-1) which extends across the embedded antisense *PCA3* gene [5,8]. Here we demonstrate that the BMCC1-1 RNA is expressed in 10/10 PCA3 positive prostate cancer tissue samples (Figure S1).

**Figure 1:**
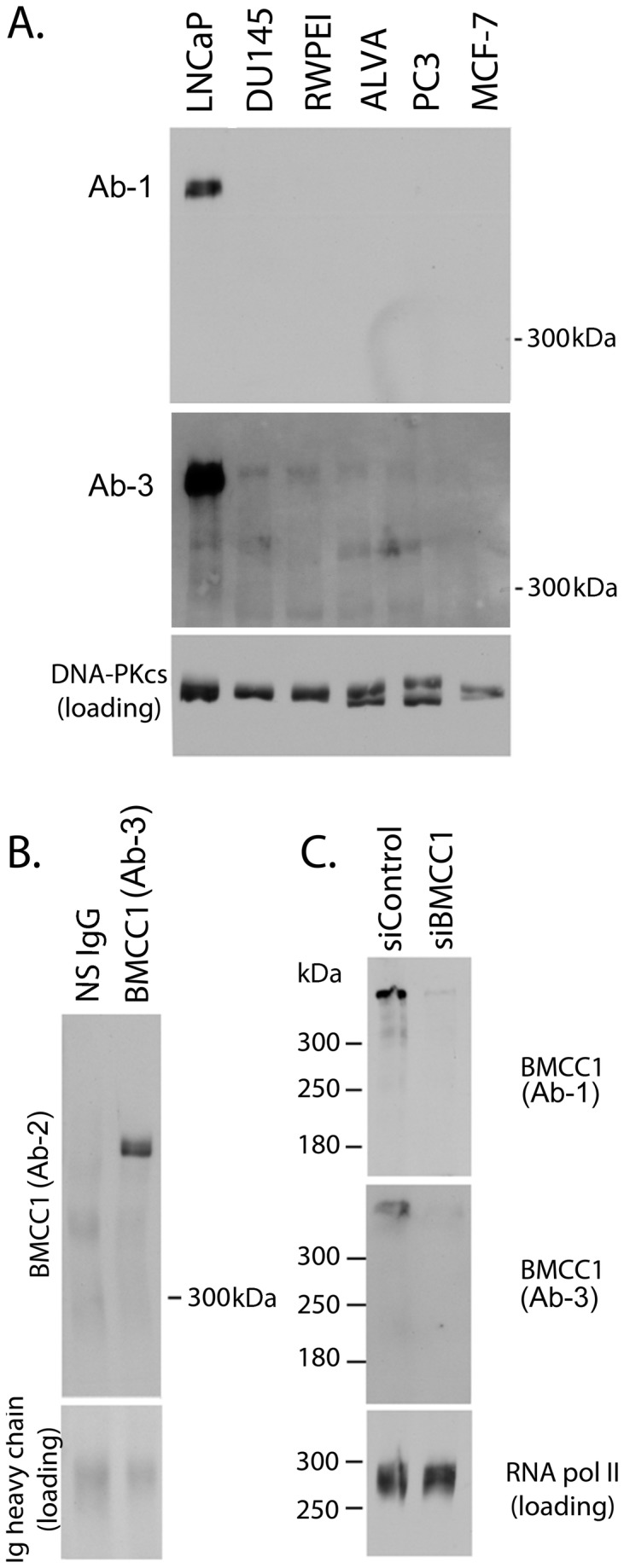
*hBMCC1* produces a 340kDa protein. **A**. **BMCC1 immunoblot of total cell extracts**. 30 µg of lysate per cell line was subjected to 5% SDS-PAGE. BMCC1 expression was detected using a rabbit antibodies raised against aa 2345 to 2705 (C-terminal, rabbit anti BMCC1, Ab-1) and a second antibody, Ab-3 that recognises the N-terminus of BMCC1. DNA PKcs was used as a loading control. **B**. **Immunoprecipitation of BMCC1 from LNCaP total lysate**. BMCC1 was immunoprecipitated by incubation of 1 mg of LNCaP lysate with 1 µg of Ab-3 for 8 h at 4°C followed by precipitation with protein G agarose. Non-specific (NS) IgG was used in place of Ab-3 in a control IP. Washed resin was eluted in SDS-PAGE loading dye and western blotted. The IgG heavy chain (loading) demonstrates equal antibody input. **C**. **Depletion of BMCC1 expression by siRNA**. LNCaP cells were transfected with BMCC1 siRNA 1730 (siBMCC1) or control siRNA (siControl). Total lysate was harvested 3 days post-transfection and BMCC1 expression analysed by western blotting with BMCC1 Ab-1 (C-terminal) and Ab-3 (N-terminal). The blot was completely stripped between sequential probes. RNA polymerase II is a loading control.

We have previously shown that the prostate cancer cell line LNCaP expresses high levels of both BMCC1-1 and PCA3 RNAs [5]. To extend these studies to endogenous BMCC1 protein, we generated several polyclonal antibodies using recombinantly expressed fragments of BMCC1 as antigens (schematic in Figure S2, cloning primers in Table S1). These antibodies were employed to demonstrate that *BMCC1* produces a >300 kDa protein in LNCaP (Figure 1). The size of this protein corresponds to that predicted from the BMCC1-1 cds and translated peptide sequence (*ie* 3088 amino acids, ~340 kDa). The 340 kDa BMCC1 protein was detected with both C-terminal Ab-1 (raised against amino acid 2345-2705) and N-terminal Ab-3 (raised against amino acid 1-369) in LNCaP cells. Expression of this protein was not detected in the PCA3 negative prostate cancer cell lines PC3, DU145, ALVA, RWPE-I (Figure 1A). BMCC1 expression was also absent from the PCA3 negative breast cancer cell line MCF7. BMCC1 was not detected in normal cells as we have previously reported [5]. The identity of the 340 kDa band was confirmed by immunoprecipitation of BMCC1 from LNCaP extracts using Ab-3, followed by Western blot detection with Ab-2 (raised against amino acid 222-276) (Figure 1B). The specificity of BMCC1 detection was further confirmed by transient depletion of BMCC1 expression by siRNA and detection with multiple antibodies. BMCC1 siRNA transfection caused a dramatic reduction in the intensity of the 340 kDa protein band detected by both C- and N- terminal antibodies Ab-1 and Ab-3 (Figure 1C). This supports the immunoprecipitation data to robustly demonstrate that *BMCC1* is a protein coding gene, producing a 340 kDa protein in the PCA3 expressing cell line LNCaP. We did not detect expression of siRNA sensitive anti-BMCC1 reactive bands at lower molecular weights, indicating that the BMCC1-1 protein is the predominant gene product in prostate cancer cells (data not shown). BMCC1 protein expression was not detected in several cell lines derived from neuroblastoma, breast and ovarian carcinoma (data not shown). BMCC1 protein was detected in 3/5 primary melanoma cell lines, and in some cases a doublet was observed (Figure S3). The identity of the bands in melanoma cell lines was demonstrated robustly by detection of BMCC1 with antibodies to different regions of the protein, Ab-1 and Ab-3. This indicates that BMCC1 could have a role in other tumour types.

Machida et al. [7] and Li et al. [12] used *in situ* hybridization of mouse embryos to show that BMCC1 RNA is expressed predominantly in neuronal tissue, and Zhou et al. [20] used PCR of mouse tissue RNA to show that at least the BCH domain is expressed in a range of tissue types. Iwama et al. [8] used PCR primers spanning the whole gene to demonstrate strong expression in the dorsal root ganglion with intermediate expression in the whole brain, spinal cord, prostate and uterus and low expression in other tissues. To address this discrepancy of constitutive versus restricted RNA expression, and to obtain additional information about protein expression and putative spliced gene products, we immunoblotted protein lysates from a panel of adult mouse tissues using BMCC1 Ab-4. In this non-tumourigenic setting we detected a strong ~80 kDa band in brain tissues, with lower expression of a smaller 72 kDa band (Figure S4). The 52kDa BMCC1s identified by Arama et al. [11] was not observed here because the antigen for Ab-4 spans amino acids 2192-2433 of BMCC1-1 and does not contain any portion of BMCC1s (see Figure S2). To confirm expression of BMCC1 in a prostate cancer tumour setting, immunohistological staining of prostate cancer tissues was performed using Ab-3. Minimal BMCC1 staining was observed in benign prostate hyperplasia glandular epithelium and stroma, with stronger staining observed in the glandular epithelium of prostate tumours (Figure S5).

### BMCC1 interacts with AP-2 in prostate cancer cells

**Figure 2:**
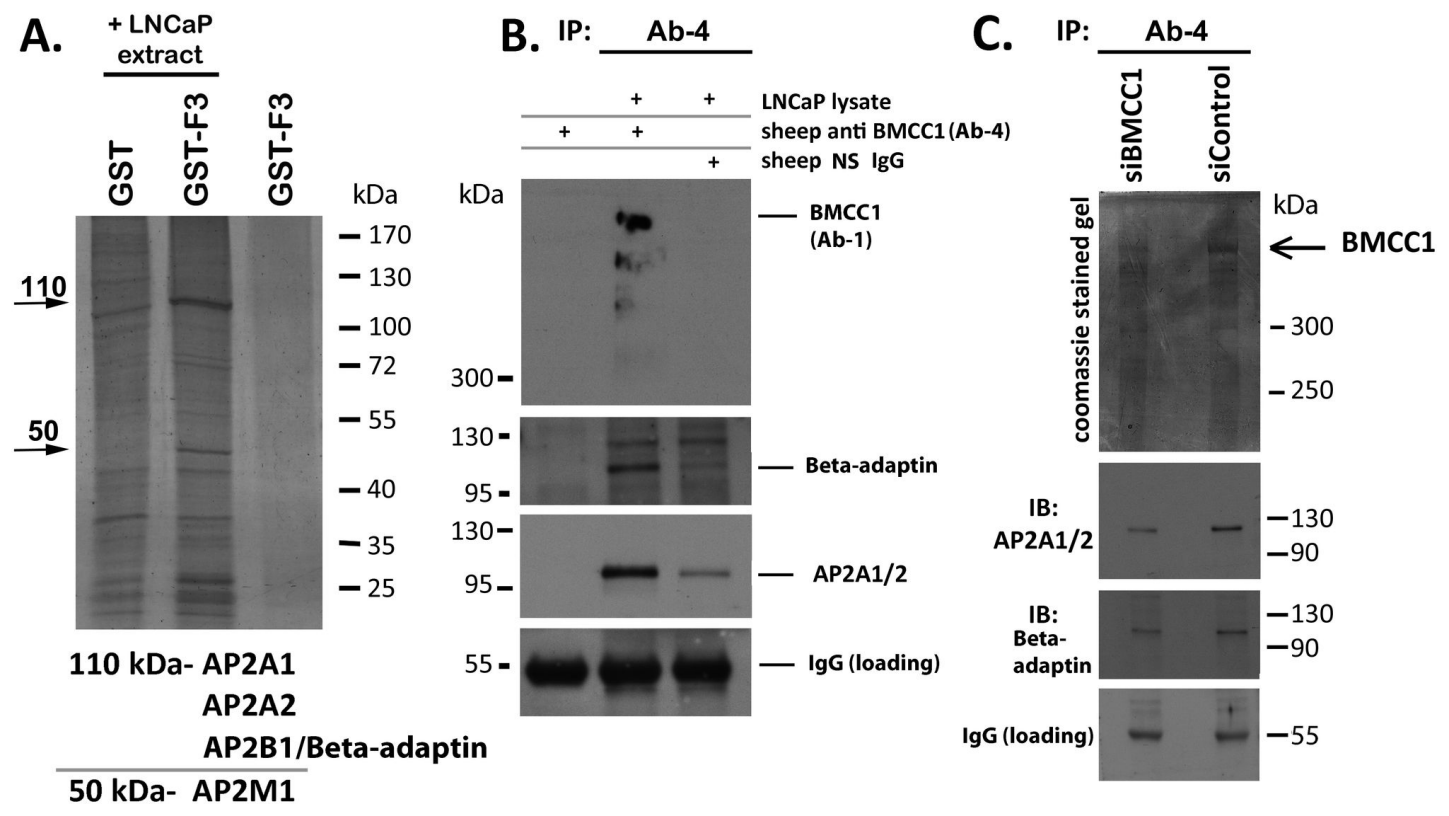
BMCC1 protein interaction mapping. **A**. **Purification of BMCC1 interacting proteins**. Crosslinked BMCC1 GST fragment 3 was used as bait to purify interacting proteins. GST crosslinked resin is a non-specific binding control. Interacting proteins were analysed by silver-stained SDS-PAGE. BMCC1-F3 interacting proteins were excised from the silver stained gel, processed and identified by MALDI TOF/TOF. Band identities are indicated. B. **Immunopurification of BMCC1 and interacting proteins**. Sheep anti-BMCC1 Ab-4 or nonspecific (NS) sheep IgG were incubated with LNCaP total lysate overnight and complexes were precipitated with protein G agarose. Immunoprecipitates were subjected to immunoblot using antibodies specific for BMCC1 (Ab-1), β-adaptin and AP2A1/2 as indicated. IgG (loading) indicates equal antibody input. C. **Immunopurification of BMCC1 and interacting proteins, using MALDI MS/MS identification**. 80% confluent T-75s of LNCaP cells were harvested 3 days post-transfection with control siRNA (siControl) or BMCC1 siRNA (siBMCC1). Clarified lysates were immunoprecipitated with Ab-4 and analysed by Coomassie stained 5% gel or western blotting duplicate 5-15% gels for AP-2A1/2 and β-adaptin. IgG (loading) indicates equal antibody input. Immunoprecipitated BMCC1 was excised from the Coomassie gel and its identity was confirmed by MALDI MS/MS (this particular Coomassie gel has been Gamma-curve adjusted for visual clarity and corresponds to MALDI data Sample A in Supp Table 4).

In order to gain insight into the cellular function of BMCC1, interacting proteins were identified by purification with BMCC1-GST affinity resins. We generated a series of overlapping BMCC1-GST clones in pGEX that cover the full-length cDNA (Table S1). Recombinant proteins were expressed and crosslinked to glutathione agarose to generate a series of affinity matricies for interacting proteins. Crosslinked GST-BMCC1 fusion resins were incubated with total LNCaP lysate to purify BMCC1 interactors. Resin associated proteins were eluted, resolved and visualised by silver stained SDS-PAGE. BMCC1 GST fragments 1-2 and 4-10 pulled down a range of proteins frequently identified as non-specific or non-functional interactors. These include eEF1γ, 40S ribosomal protein, GRP75 and GRP78 (data not shown). BMCC1 GST F3 (amino acids 575-904) precipitated specific bands at 50 kDa and 110 kDa (Figure 2A). The 50 kDa and 110 kDa interacting bands were excised and identified by mass spectrometry (MALDI MS/MS on a Bruker Ultraflex III). A summary of Mascot search data is shown in Table S2. This revealed that BMCC1-F3 interacts with several members of the Adapter Related protein Complex 2 (AP-2). The AP-2 complex is an endosome associated heterotetramer consisting of three different constitutive components (AP-2S1, AP-2M1 and AP-2B1/β-adaptin) and one of two mutually exclusive components (AP-2A1 or AP-2A2) [21]. The 50 kDa BMCC1-F3 interacting protein was identified as AP-2M1, based on two separate identifications with a total of 8 non-redundant peptides (Table S2). The 110 kDa BMCC1 interacting band contained a mixture of three high molecular weight AP-2 complex members: AP-2A1, AP-2A2, and AP-2B1 (Table S2). AP-2B1 was identified in two samples with a total of 5 unique peptides. The homology between AP-2A1 and AP-2A2 makes individual unique identifications complex (Figure S6). AP-2A1 was identified in 3 samples with a total of 6 peptides that do not correspond to AP-2A2. AP-2A2 was identified in one sample with one peptide not attributable to AP-2A1. In these samples, three individual peptides could have corresponded to either AP-2A1 or AP-2A2 and cannot be attributed with certainty to one or the other due to sequence identity (Table S3). No computationally identifiable domains could be attributed to this region, so functional interaction mapping of BMCC1 has identified a novel protein–protein interaction motif. The interaction between BMCC1 and the AP-2 complex was confirmed by co-immunoprecipitation. BMCC1 was immunoprecipitated from LNCaP lysates and the resulting interacting proteins were eluted and analysed by Western blotting (Figure 2B) revealing that endogenous BMCC1 interacts with β-adaptin and AP-2A1/2 (Figure 2B). The endogenous interaction between BMCC1 and the AP-2 complex was further substantiated by BMCC1 immunoprecipitation from lysates of cells which had been BMCC1 depleted with siRNA. A 340 kDa band was detected on the high molecular weight resolving Coomassie stained gel (Figure 2C). This band was of reduced intensity in the immunoprecipitate from BMCC1 depleted cells compared to the control siRNA cells. Although immunoprecipitation is not a quantitative approach, this reduced intensity guided band selection for subsequent MS analysis. The identification of this band as BMCC1 was confirmed by MALDI-TOF/TOF of tryptic peptides from the excised gel slice. Importantly, the extreme BMCC1 N-terminal peptide TEFNYFTETR, not present in the truncated isoform (BMCC1-3) reported by Machida et al. [7] was identified among these (Table S4). This provided *de novo* evidence of BMCC1-1 protein expression in LNCaP cells. The interaction profile of BMCC1 was examined by Western blotting these immunoprecipitates for AP-2A1/2 and β-adaptin. Consistent with the reduced level of BMCC1 protein in the siBMCC1 immunoprecipate, reduced signals for AP-2A1/2 and β-adaptin were detected in the precipitate from siBMCC1 lysate than control lysate. This provided further evidence that these proteins specifically interact with BMCC1 (Figure 2C). We have not explored the interaction between BMCC1 and AP-2M1 by immunoprecipitation due to the size similarity between AP-2M1 and the IgG heavy chain, and the lack of availability of quality AP-2M1 antibodies. However, AP-2M1 is a constitutive component of the AP-2 complex and BMCC1’s demonstrated interaction with both A/α and B/β AP-2 subunits indicates that AP-2M1 would also be present.

### BMCC1 co-localises with endocytic vesicles at various stages of biogenesis

**Figure 3:**
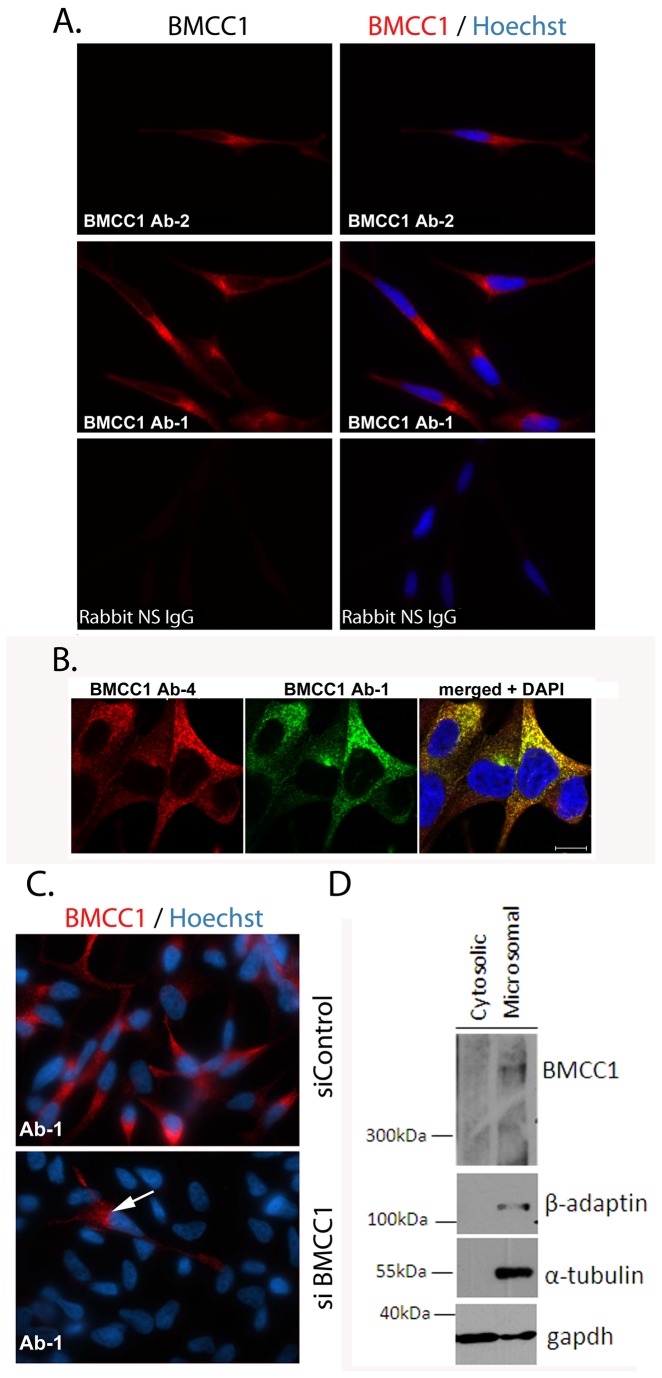
Cellular distribution of BMCC1. A. **BMCC1 distribution in LNCaP**. BMCC1 was labelled by immunostaining with rabbit anti-BMCC1 antibodies to the C- or N- (Ab1, Ab2) terminus or non-specific (NS) rabbit antibody and detected with donkey anti-rabbit Alexafluor 594. Cells were counterstained with Hoechst. Staining was analysed by wide-field fluorescent microscopy (x63). B. **High resolution imaging of BMCC1 in LNCaP**. Fixed LNCaP were incubated with a rabbit anti-BMCC1 (Ab-1) and a sheep anti-BMCC1 (Ab-4). Cells were stained with donkey anti-rabbit Alexafluor 488 and donkey anti-sheep Alexafluor 594. Staining was analysed by confocal fluorescent microscopy. C. **Ablation of BMCC1 immunostaining by siRNA**. LNCaP were transfected with BMCC1 siRNA 1730 (siBMCC1) or control siRNA (siControl) using Lipofectamine RNAiMAX. Cells were fixed 3 days post transfection and stained with rabbit anti-BMCC1 (Ab-1) and detected using donkey anti-rabbit Alexafluor 594. D. **Subcellular fractionation of LNCaP**. LNCaP cytoplasmic extract was generated by dounce homogenization in hypotonic solution and sedimentation of nuclei. Microsomes were precipitated from the recovered cytoplasm by ultracentrifugation. BMCC1 compartmentalization was analysed by western blotting the resulting microsomal and cytosolic fractions using antibodies against BMCC1 (Ab-4) (5% gel), β-adaptin and α-tubulin (microsomes) and GAPDH (cytosolic) (duplicate 12% gel).

The subcellular distribution of BMCC1 in LNCaP was investigated using immunofluroescence with N- and C- terminal antibodies. BMCC1 displayed granular cytoplasmic staining with both antibodies (Figure 3A). High resolution confocal immunofluorescent analysis of BMCC1 localisation using two different BMCC1 antibodies displayed strong overlap, indicating that both antibodies detect the same protein (Figure 3B). This analysis revealed that BMCC1 is present in small vesicles, which are present throughout the cytoplasm and particularly concentrated in the juxta-nuclear region. The specificity of BMCC1 immunostaining was further confirmed by depletion of BMCC1 immunostaining in LNCaP cells transfected with BMCC1 siRNA (Figure 3C). Control transfected cells have a range of BMCC1 expression levels by immunostaining (Figure 3C, upper panel). Consistent with strong BMCC1 depletion observed by western blotting (Figure 1C), BMCC1 stained cells are infrequent after transfection with siBMCC1 (Figure 3C, arrow) and most cells are completely negative. BMCC1 immunostaining of aphidicolin synchronized LNCaP cultures revealed that the expression and distribution of BMCC1 is independent of cell cycle phase (Figure S7). The distribution of BMCC1 throughout the cytoplasm was further investigated by separation of cytoplasmic vesicles from whole cytoplasm, yielding cytosolic and microsomal fractions. BMCC1 was detected exclusively in the microsomal fraction, indicating that BMCC1 is strongly vesicle associated (Figure 3D).

**Figure 4:**
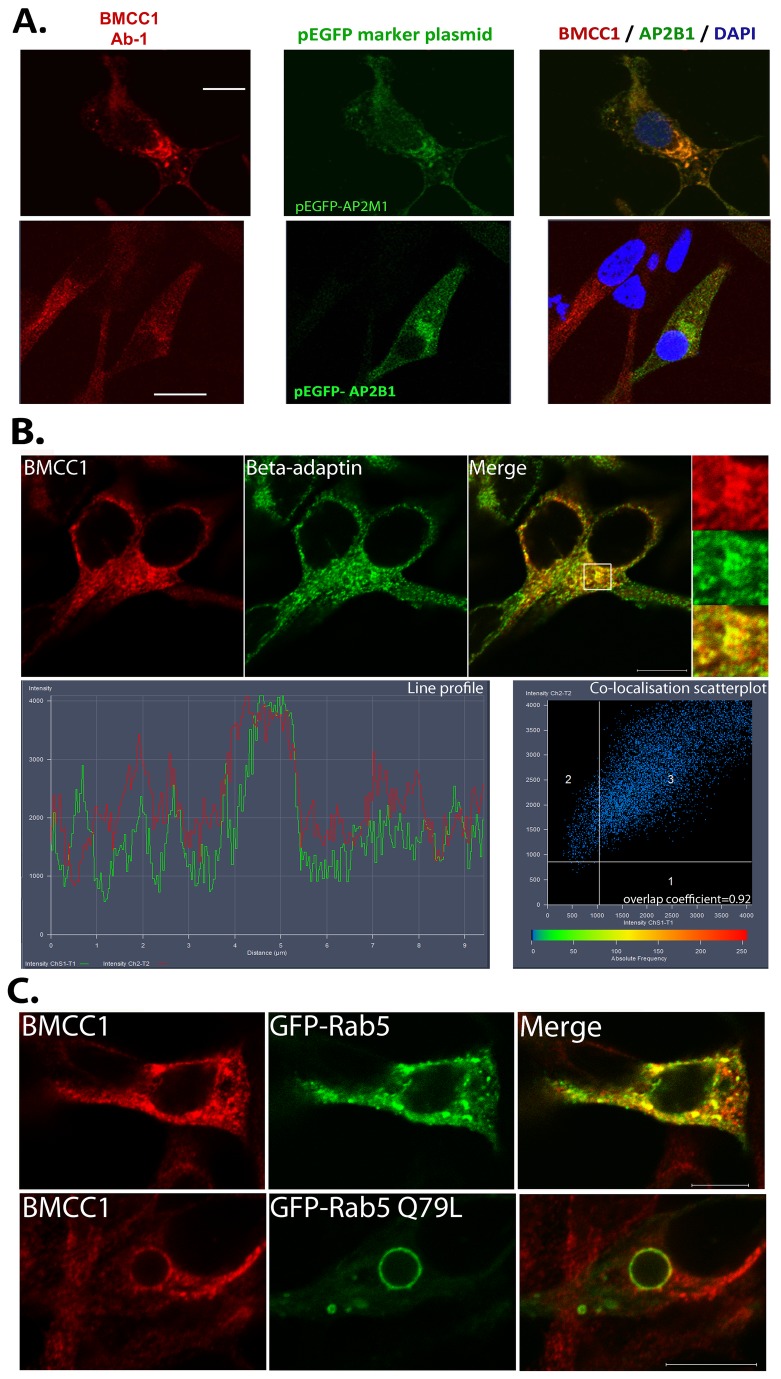
BMCC1 is endosomal. A. **Co-localisation of BMCC1 and endosomal reporter proteins**. LNCaP were transfected with pEGFP-AP-2M1 or pEGFP-AP-2B1. BMCC1 protein was detected using rabbit anti-BMCC1 (Ab-1) and donkey anti-rabbit Alexafluor 594 72 h post-transfection. Localisation was analysed by confocal microscopy (100 x objective). Scale bars are 10 µm. B. **Co-localisation of BMCC1 with β-adaptin**. LNCaP were fixed and stained with sheep anti- BMCC1 (Ab-4) and mouse anti-β-adaptin. Proteins were detected with donkey anti-sheep Alexafluor594 and donkey anti-rabbit Alexafluor488. Cells were counterstained with DAPI and analysed by confocal microscopy (63 x objective). Scale bar is 10 µm. The line profile shows overlapping fluorescence signals for BMCC1 and β-adaptin and was generated by drawing a horizontal line through the boxed area in the perinuclear region of the cell using Zen 2011 analysis software. A circle was drawn around the same area to generate the co-localisation scatterplot and overlap coefficient data using Zen 2011 analysis software (x axis = green fluorescence, y axis = red fluorescence). C. **Co-localisation of BMCC1 and Rab5**. Cells were transfected with pEGFP-Rab5a (upper panel) or pEGFP- Q79L Rab5a (lower panel). BMCC1 was detected using rabbit anti-BMCC1 (Ab-1) and donkey anti-rabbit Alexafluor 594, 24h post-transfection. Stained cells were mounted in Prolong Gold containing Dapi and examined by confocal microscopy (63x). Scale bars are 10 µm.

The granular cytoplasmic distribution of BMCC1 and its constitutive interaction with the AP-2 complex led us to explore co-localisation between BMCC1 and AP-2 proteins. LNCaP cells were fixed and stained for BMCC1 three days after transfection with AP-2M1 or AP-2B1 pEGFP constructs or vector control. Substantial co-localisation was observed between BMCC1 and AP-2M1 and AP-2B1 (Figure 4A). AP-2M1-GFP displayed a cytoplasmic tubulovesicular structure with small vesicles associated with the cell periphery and larger structures in the peri-nuclear region. BMCC1 was associated with the larger juxta-nuclear AP-2M1 labelled vesicles, but was less abundant in the small peripheral vesicles closely associated with the plasma membrane (Figure 4A, upper panel). BMCC1 staining of AP-2B1-GFP transfected cells revealed that BMCC1 and AP-2B1 partially co-localize and are present on the same vesicles (Figure 4A, lower panel). The substantial difference in localisation of BMCC1 and AP-2 complex members between AP-2B1 and AP-2M1-GFP transfected cells probably represents aberrant localisation of AP-2M1-GFP. The high level of exogenous expression required to detect the GFP proteins by confocal microscopy could cause abnormal localisation or changes in endocytic function [22]. As such these data support the interaction/co-localisation of BMCC1 and AP-2 but occur under conditions of abnormal vesicular morphology. Therefore the localisation of BMCC1 and the AP-2 complex was subsequently explored using β-adaptin immunostaining of endogenous protein. Co-staining with anti-BMCC1 and β-adaptin confirmed the juxta-nuclear co-localisation of BMCC1 and the adaptor related protein complex (Figure 4B, upper panel). A line profile through the boxed region, in the peri-nuclear region of the cell, shows overlapping fluorescence signals for BMCC1 and β-adaptin. A circle was drawn around this region to generate the co-localisation scatterplot and an overlap coefficient of 0.92 was calculated using Zen 2011 analysis software (x axis = green fluorescence, y axis = red fluorescence) (Figure 4B, lower panel). Taken together, the co-staining and GFP co-localisation data indicate that the AP-2 complex and BMCC1 coat endosomes in the cell periphery and juxta-nuclear regions. BMCC1 appears to be associated weakly with the early, more peripheral endosomes and more strongly with tubulovesicular structures adjacent to the nucleus.

In order to further characterise the specific vesicle type BMCC1 is associated with, we also tested BMCC1 co-localisation with the small GTPase Rab5. Rab5 is present on primary endocytic structures but is most abundant on early endosomes where it regulates the activity and recruitment of downstream effectors prior to additional homotypic and heterotypic fusion [23]. LNCaP cells were transfected with GFP-Rab5a prior to staining with anti-BMCC1. Rab5a-GFP transfected cells display substantial areas of co-staining between BMCC1 and Rab5a (Figure 4C, upper panel). We also employed a GTP-ase deficient form of Rab5 (Q79L) which induces formation of enlarged early endosomes and also enlarged multivesicular endosomes [24]. LNCaP cells transfected with Rab5 Q79L showed large ring-like structures that co-stained for BMCC1 and Rab5a (Figure 4C, lower panel). While Rab5a and BMCC1 co-localisation was evident, many vesicles were positive for one protein or the other. This indicates that BMCC1 is present on a subset of sorting endosomes only and constitutes more than one vesicle type. As further proof of the presence of BMCC1 in early sorting endosomes we showed minor co-localisation and juxta-position of BMCC1 with the early endosome marker EEA1 (Figure S8). Many BMCC1 positive vesicles were EEA1 negative, again supporting the presence of BMCC1 on other vesicle types/at other stages of endosomal biogenesis. EEA1 is specifically and strongly associated with the early sorting endosome and is not observed in other compartments or at other stages of endocytic processing, in direct contrast to most other endocytic proteins, including Rab5 [25]. The stronger overlap between BMCC1 and Rab5 staining could indicate that many EEA1 negative BMCC1 vesicles could still be Rab5 positive.

**Figure 5:**
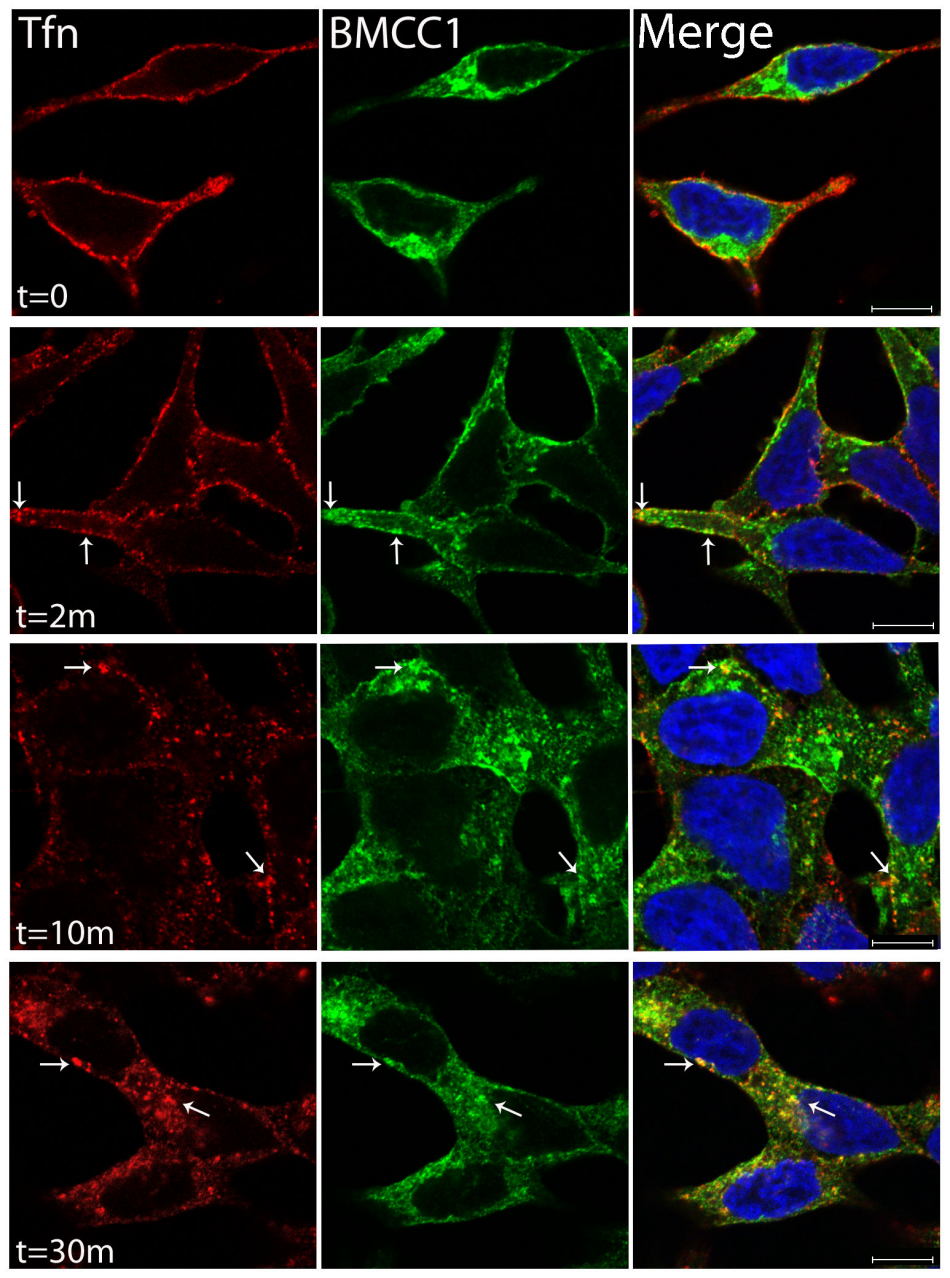
BMCC1 and transferrin co-localisation. **Co-localisation of BMCC1 and pulse-chase labelled transferrin**. LNCaP cells were coated with Alexafluor594 conjugated transferrin in ice-cold serum free media for 30 min and fixed at the indicated chase times. Cells were BMCC1 immunostained with Ab-4, detected with Alexafluor488 and mounted in Prolong Gold with Dapi (63x confocal). Arrows indicate areas of BMCC1 and transferrin co-localisation. Scale bars are 10 µm.

We next explored co-localisation of BMCC1 with Alexafluor594 conjugated transferrin (Tf). The transferrin-receptor complex (Tf-R) is processed through early endosomes and targeted to the recycling compartment of the endosome. In pulse-chase experiments, labelled Tf did not immediately co-localise with BMCC1. BMCC1 began to show some co-localisation with conjugated Tf by the 2 min chase time point and strong co-localisation by 30 min (Figure 5). Co-localisation of BMCC1 with Tf at the early time point is consistent with the presence of BMCC1 on early sorting endosomes, while the strong co-localisation at later stages of uptake suggests that BMCC1 persists on recycling endosomes.

### BMCC1 and trafficking

**Figure 6:**
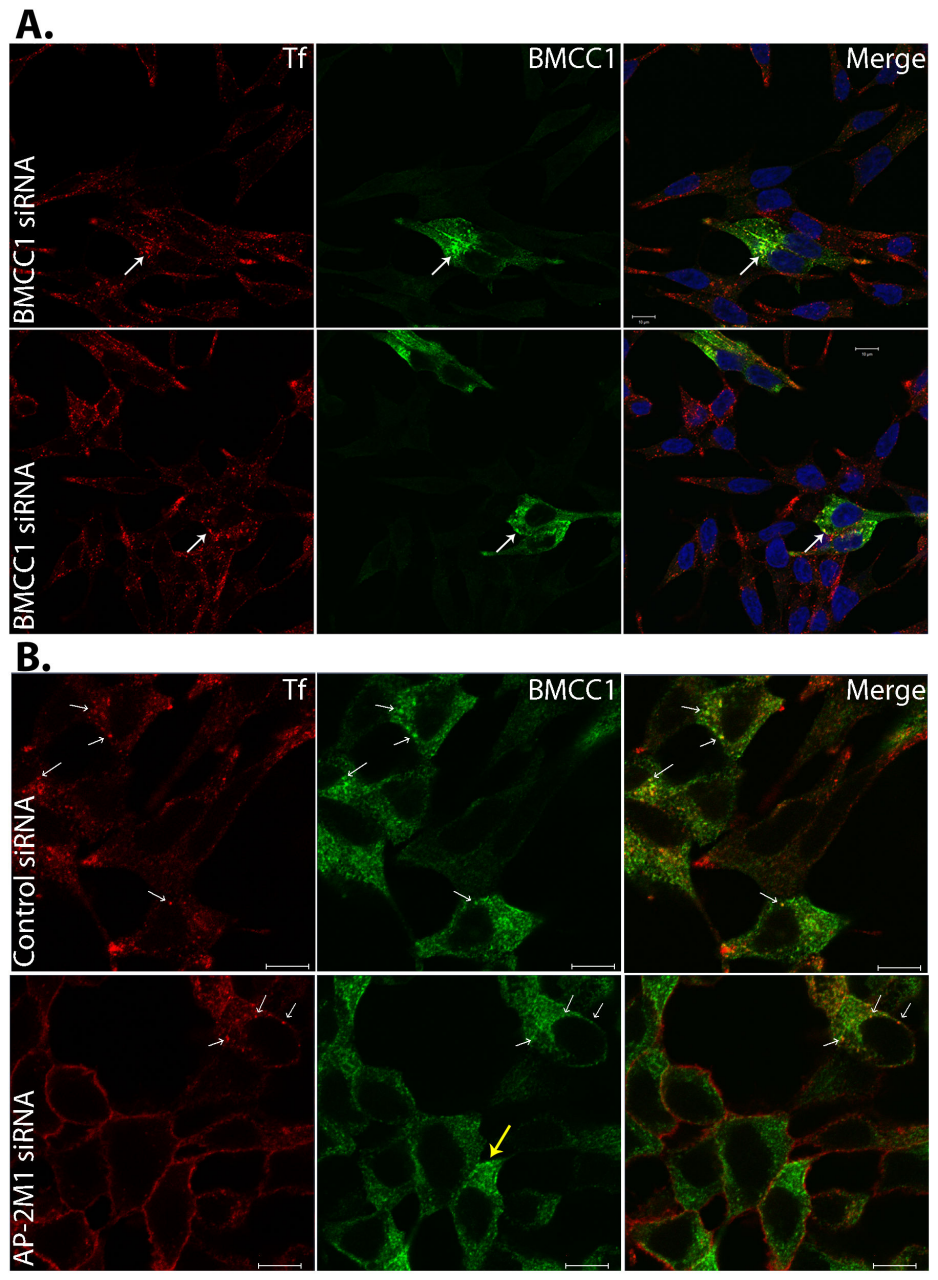
BMCC1 and vesicle trafficking. **A**. **BMCC1 is not required for transferrin uptake**. LNCaP cells were tranfected with siRNA 1730 to knockdown BMCC1. Cells were subjected to a 10 min pulse with Alexafluor594 conjugated transferrin, 48 h post-transfection. Cells were then rinsed with ice-cold PBS to remove excess conjugate before fixation. Cells were BMCC1 immunostained with Ab-4, detected with Alexaflour 488 and mounted in Prolong Gold with Dapi (63x confocal). Scale bars are 10 µm. B. **BMCC1 vesicular localisation is independent of AP-2**. LNCaP cells were subjected to two subsequent 48 h transfections with siRNA s3112 to knockdown AP2-M1. Cells were then subjected to a 10 min pulse with Alexafluor594 conjugated transferrin. Cells were then rinsed with ice-cold PBS to remove excess conjugate before fixation. Cells were BMCC1 immunostained with Ab-4, detected with Alexafluor 488 and mounted in Prolong Gold with Dapi (63x confocal). White arrows indicate areas of BMCC1 and transferrin co-localisation, while the yellow arrow highlights a cell displaying typical BMCC1 vesicular localisation in the absence of functional AP-2. Scale bars are 10µm.

Receptor mediated endocytosis of transferrin is heavily dependent on the AP-2 complex. Depletion of the constitutive µ/M subunit causes a ~90% reduction in the efficiency of endocytosis of the transferrin receptor, but does not affect internalization of EGF or LDL [26]. This suggests that while AP-2 is necessary for endocytosis of some receptors, other cargo molecules are able to utilize an alternative endocytic mechanism. The interaction and co-localisation of BMCC1 with the AP-2 complex, combined with the localisation of BMCC1 to functional Tf transporting vesicles points to a possible role for BMCC1 in vesicle trafficking. To begin to address this, we examined the effects of BMCC1 depletion on Tf uptake. In cells transfected with control siRNA, punctate staining of internalised Tf was observed following a 10 min uptake, and co-localisation with BMCC1 was evident (data not shown). In cells treated with BMCC1 siRNA, normal BMCC1 staining was observed in ~20% of cells, which is consistent with the knockdown efficiency of 70-80% we measured by qPCR (qPCR data not shown). In these cells, co-localisation of BMCC1 and Tf was evident (Figure 6A, white arrows). In cells with strongly depleted levels of BMCC1, Tf levels, uptake and distribution remained undisturbed (Figure 6A). Further analysis of BMCC1 roles in Tf trafficking were impeded by the fact that membrane-bound Tf must be removed by acid wash in order to measure recycling efficiency/clearance. Unfortunately, acid rinses distorted and reduced BMCC1 staining (data not shown) making it impossible to measure recycling in BMCC1 knockdown cells only.

We used another approach to further investigate BMCC1 roles in trafficking. The AP-2 complex is a protein hub central to the formation of endosomes. It interacts with the cell membrane and cell membrane-associated cargo proteins as well as with accessory proteins which are essential for invagination and budding of endosomes [21]. Importantly, knockdown of AP-2 has been shown to moderately disrupt the vesicular localisation of accessory proteins [26,27]. Moreover, specific cargo (Tf) uptake is dependent on AP-2 [26]. Since BMCC1 interacts with AP-2 and co-localises with AP-2 and Tf, we sought to determine if AP-2 is required for BMCC1 vesicular localisation and/or formation. To deplete the AP-2 complex in LNCaP cells we used AP-2M1 siRNA. We attained an average of 88% knockdown efficiency for AP2-M1 in these experiments (measured by qPCR, data not shown) and functional depletion of the AP-2 complex was assessed via the inability of cells to uptake labelled Tf. Cells transfected with control siRNA showed normal punctate staining for internalised Tf (red), and co-localisation with BMCC1 (green) was once again observed (Figure 6B, upper panel, white arrows). Following AP-2M1 siRNA knockdown, Tf uptake was inhibited and it remained trapped at the membrane (red staining) which indicated successful depletion of functional AP-2 complexes. In these depleted cells, neither BMCC1 vesicular localisation nor number of vesicles was apparently disturbed (Figure 6B, lower panel). The yellow arrow highlights one cell with functionally depleted AP-2 complexes and typical BMCC1 staining. These data suggest that even though BMCC1 and AP-2 interact and co-localise, BMCC1 is not an archetypal AP-2 accessory or cargo protein and BMCC1 vesicle formation does not depend on AP-2. Instead, BMCC1 is a novel endosomal protein which must interact with AP-2 during post-endocytic uptake trafficking stages.

## Discussion

This study provides persuasive evidence for the expression of a high molecular weight form of BMCC1 protein in prostate cancer and melanoma cell lines. We established BMCC1 as an AP-2 interacting protein that is localised to endocytic vesicles. The results concur with and extend earlier expression studies on full-length mRNA and protein for BMCC1. Iwama et al. [7,8] cloned a full-length cDNA and used immunoblotting to identify a BMCC1 (PRUNE2) 338kDa protein. Here we developed several antibodies to different regions of BMCC1 and used these in combination with siRNA and MALDI MS/MS to provide conclusive evidence for the expression of the high molecular weight BMCC1 protein. Doubt existed on the extent of tissue expression of BMCC1 protein isoforms since initial studies were based on PCR [6,20]. Machida et al. [7] used semi-quantitative RT-PCR to describe widespread expression of BMCC1 in tissues including the nervous system and variable expression in a variety of tumour cell lines. Use of *in situ* hybridization by Li et al. [9] indicated that expression was high in developing neuronal tissues. Arama et al. [11] demonstrated expression of a 52 kDa BMCC1s protein in neurones, which a follow-up study by Li et al. [12] supports. The results described here reveal BMCC1 protein expression in the cerebellum and cerebrum of adult mouse tissue lysates, with the predominant band at approximately 80 kDa. While both Li et al. [12] and Arama et al. [11] found that the predominant BMCC1 protein expressed in their brain samples was 52 kDa, both studies also detected a band of approximately 75 kDa. Differences in the relative intensity of detected bands in our studies may be due to the differing ages of our animals and antibody target regions. Subsequent characterisations of tissue specific expression could utilize the RNA segment corresponding to these antibody targets as starting points for RACE-PCR.

One approach to understand the function of BMCC1 is to identify interacting proteins. Soh and Low [6] identified BNIPXL as a putative BNIP2 and Cdc42GAP (BCH) domain protein, 769 amino acids in length, corresponding to a C-terminal region of the BMCC1 protein described here. They showed that a recombinant form of this protein interacted with and inhibited the activity of the small GTPase, Rho A. Signalling through RhoA regulates actin dynamics, cellular morphogenesis and motility [28]. More specifically, signalling through this protein is linked to neuronal migration, growth cone collapse, dendritic branching, neurodegeneration and cancer [29,30]. BMCC1/PRUNE2 was identified in a large scale purification of GTP and 8-oxo-GTP binding proteins [8]. The same study identified T-cell activation Rho GTPase activating protein as a binding partner of BMCC1/PRUNE2, albeit with a low confidence score. The latter data suggest that BMCC1 (PRUNE2) may be interacting with GTP-signalling proteins to alter their activity. Arama et al. [11] recently found that the 52 kDa brain specific isoform of BMCC1 localized to microtubules and interacted with the microtubule stabilizing protein MAP6/STOP [31]. This BMCC1 isoform blocked the interaction of MAP6 and microtubules, which inhibited microtubule stabilization and changed cell morphology [11]. They showed punctiform localisation of this 52kDa BMCC1 isoform to microtubules, but the striking vesicular localisation presented in our study was not evident [11]. This is not surprising given that the AP-2 interaction domain characterised herein is not present in the shorter brain-specific isoform. In a recent follow-up study, BMCC1s was shown to interact with kidney-type glutaminase, a mitochondrial enzyme which converts glutamine to glutamate in neurons [32]. Over-expression of BMCC1s induced re-localisation of mitochondrial KGA to the cytoplasm. The authors proposed a mechanism whereby BMMC1s indirectly influences trafficking of KGA through destabilisation of microtubule networks [32].

In the present study, we employed GST-pulldowns to identify several members of the endosome-associated AP-2 complex as binding partners for BMCC1 with reproducible, very high confidence scores. The AP-2 complex is an adaptor in clathrin-mediated endocytosis [33]. This complex associates with phosphoinositides, clathrin and other membrane proteins to co-ordinate cargo recruitment for internalisation, as well as accessory proteins which mediate the structural formation of endsomes [21]. The incoming cargo is received by early endosomes which act as a sorting station in the endocytic pathway [34]. In this study, we showed that BMCC1 was present in the microsomal fraction of the cytoplasm and localised to cytoplasmic granules. Initial studies revealed that BMCC1 protein was cytoplasmic in tissue sections but failed to provide further detail regarding isoform expression or more specific localisation [7,8]. Here we showed that members of the AP-2 complex co-localised with BMCC1 in cytoplasmic granules, identifying them as clathrin-coated pits, vesicles or endosomes. This was verified using three different antibodies against BMCC1. BMCC1 also showed co-localisation and juxtaposition with early sorting endosome markers Rab5 and EEA1 [24], as well as strong co-localisation with the recycling endosome marker Tf.

We have shown in this study that AP-2 is not required for BMCC1 localisation to cytoplasmic vesicles, which would suggest that BMCC1 is not an archetypal AP-2 accessory protein. While AP-2 is pivotal at the most early stages of endocytic uptake of Tf and our data show localisation of BMCC1 to early Rab5-positive endocytic vesicles, we saw that BMCC1 knockdown does not affect Tf uptake. Moreover, our Tf localisation data seem to indicate that BMCC1 persists and becomes more prominent in the recycling endosome. Vesicular trafficking is a highly complex biological process, and many proteins appear to play multi-functional roles along different stages of the pathway. Of particular note, while AP-2 has traditionally been implicated in endocytic uptake at the plasma membrane, it has more recently been shown to affect post-endocytic trafficking and specifically to promote recycling over degradation [35]. This certainly fits with the perinuclear localisation of various AP-2 isoforms observed in this study. Given the perinuclear co-localisation of BMCC1 and AP-2 and the strong co-localisation between Tf and BMCC1, it would appear that the BMCC1-AP2 interaction occurs during post-endocytic uptake trafficking and targeting stages. It is thus conceivable that BMCC1 plays roles in specific cargo transfer from one endocytic sub-stage to the next or it may affect pathways which determine cargo destination.

In summary, we have demonstrated that BMCC1 produces a 340 kDa protein in select prostate cancer and melanoma cell lines. Comprehensive protein interaction mapping revealed that BMCC1 interacts and co-localises with the AP-2 endocytic scaffold complex in perinuclear regions of the cell. We also observed strong co-localisation with internalised Tf at later pulse-chase time points, which is indicative of the recycling endosome. We have shown that BMCC1 is not required for Tf uptake and that BMCC1 vesicular localisation is independent of AP-2. Further studies will now be required to ascertain the exact role of BMCC1 in post-endocytic trafficking. We have previously shown that PCA3 is embedded in an intron of BMCC1 [5]. The PCA3 gene is over-expressed in prostate cancer and has been shown to be a valuable biomarker for this disease [4]. The vesicular localisation of BMCC1described here provides greater insight as to how this protein functions. It is possible that BMCC1 plays an important role in some aspect of intracellular trafficking which may in turn impact on cell behaviour. Understanding the relationship between PCA3 and BMCC1 will provide additional information on how these genes function in prostate cancer.

## Supporting Information

Figure S1 **BMCC1 RNA expression.** A. **Expression of BMCC1-1**. Expression was detected in cDNA from prostate cancer biopsies using primers in exons 6 and 7. PCA3 was detected in the same samples using a forward primer in exon 1 and a reverse primer across the exon 1/3 junction. Beta-2-microglobulin was amplified as a cDNA quality and input control. The cDNA control band for Beta-2-microglobulin is primer dimer.
(TIF)

Figure S2 **Schematic of BMCC1 gene structure and predicted protein domains.** BMCC1 exon structure and protein domains were collated using the online tools ExDom, Exon-Intron Graphic Maker (wormweb.org) and Interpro: protein sequence analysis and classification. BMCC1 isoform 1 (BMCC1-1) RNA is depicted with reference to the smaller isoforms 2-4, BMCC1s and olfaxin (NCBI accession numbers are noted). The obsolete BMCC1-2 is included for illustrative purposes. The cDNA regions cloned for recombinant antigen expression and antibody production, as well as AP-2 pull-down (AP-2 interacting region) are indicated. The siRNA target region is also indicated. Note that all line depictions include intronic sequences and as such do not reflect translated peptide/protein size.
(TIF)

Figure S3 **Expression of BMCC1 in LNCaP and melanoma cell lines.** Total cell lysates were generated from the primary melanoma cell line A11, D11, D28, D33 and D38. 30 µg of total lysate from each cell line was subjected to SDS-PAGE and western blotting with rabbit anti-BMCC1 antibodies (Ab-1, Ab3). DNA-PKcs is shown as a loading control.
(TIF)

Figure S4 **Tissue expression profiling.** BMCC1 protein expression was examined in lysates from a healthy 12 week old male C57/B6 mouse. Tissues were harvested and lysed in MCLB without haemolysis, and 100 µg of clarified lysate from each tissue was resolved by SDS-PAGE on 5% or 5 to 15% gels. Proteins were detected by western blotting or Coomassie staining as indicated.
(TIF)

Figure S5 **Immunohistological staining for BMCC1 in prostate cancer tissues.** Benign prostatic hypertrophy (BP) (Panels A-C) and prostatic adenocarcinoma (PCa) (Panel D grade 3, Panel E grade 4, Panel F grade 5) tissue sections were incubated with buffer only (Panel A) or BMCC1 Ab-3 (Panels B–F) and detected using streptavidin-biotin-peroxidase immunostaining with diaminobenzidine. Size bar is 100µm (A, E, F) or 50µm (B, C, D).
(TIF)

Figure S6 **Alignment of AP-2A1 and AP-2A2.** AP-2A1 and AP-2A2 protein sequences obtained from the NCBI were aligned using Clustal W (default settings). Peptides identified by MALDI MS/MS in our purification of BMCC1 interactors are indicated, identified by sample number- peptide number with summary statistics for each peptide in Supp Table 2.
(TIF)

Figure S7 **Expression of BMCC1 throughout the cell cycle.** A. **Sychronised LNCaP cells with a single aphidicolin block (5 µg/mL) for 36 h**. Cells were released by washing in drug-free media and fixed at intervals after aphidicolin removal. Fixed coverslips were kept in PBS at 4 °C until all time points had been collected. Cells were then stained for BMCC1 (rabbit Ab-1 antibody) and detected with donkey anti-rabbit Alexafluor488. Cells were counterstained with Hoechst, mounted in moviol and analysed on a wide-field microscope (x63 objective). B. **Inhibition of S-phase progression assessed by BrdU incorporation**. Normally cycling or aphidicolin blocked cells were incubated with 100 µM BrdU for 2 h in growth media before fixation. BrdU incorporation was detected in fixed, alkaline denatured cells using rat anti-BrdU and donkey anti-rat Alexafluor 488.
(TIF)

Figure S8 **BMCC1 colocalisation and juxtaposition with early endosomal marker EEA1.** LNCaP cells were stained for sheep anti- BMCC1 and mouse anti- EEA1, and detected with anti-sheep Alexafluor594 and anti mouse- Alexafluor 488. The white arrow highlights an area of clear co-localisation between the two proteins and the yellow arrow highlights an EEA1-negative BMCC1-positive vesicle. Scale bar is 10 µm.
(TIF)

Table S1 Cloning, PCR and siRNA primer sequences.
(TIF)

Table S2 MALDI TOF/TOF data summary- BMCC1 interactions.
(TIF)

Table S3 Attribution of identified peptides between high-homology AP-2A1 and AP-2A2.
(TIF)

Table S4 MALDI TOF/TOF data summary- BMCC1 primary sequencing.
(TIF)
