# Supplementary figures and images for: BMCC1 Is an AP-2 Associated Endosomal Protein in Prostate Cancer Cells
Source: PLoS One. 2013 Sep 6;8(9):e73880. doi: 10.1371/journal.pone.0073880 (PMC3765211; doi:10.1371/journal.pone.0073880)

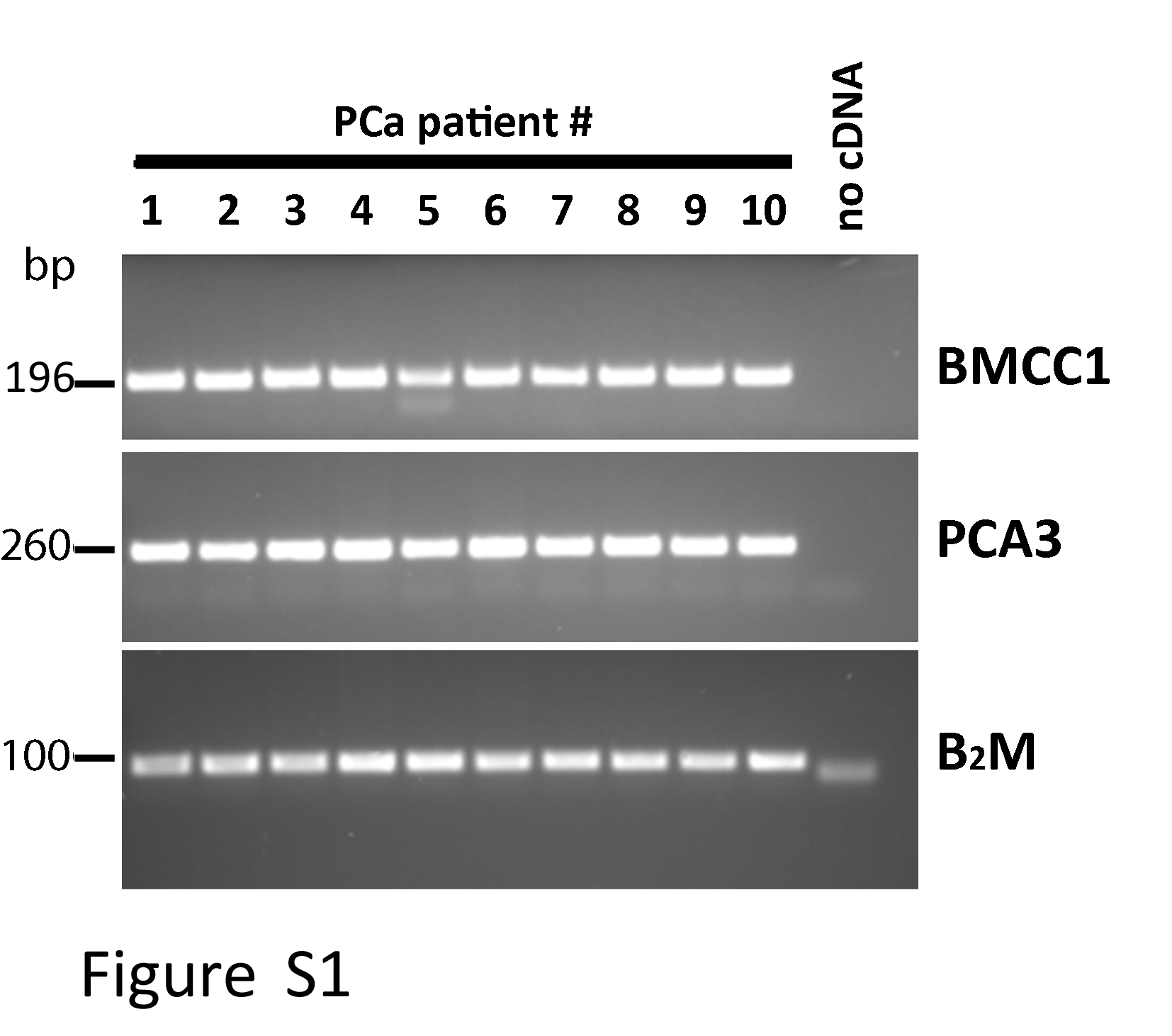

Supplement: Figure S1 — BMCC1 RNA expression. A. Expression of BMCC1-1. Expression was detected in cDNA from prostate cancer biopsies using primers in exons 6 and 7. PCA3 was detected in the same samples using a forward primer in exon 1 and a reverse primer across the exon 1/3 junction. Beta-2-microglobulin was amplified as a cDNA quality and input control. The cDNA control band for Beta-2-microglobulin is primer dimer. (TIF) [file pone.0073880.s001.tif]

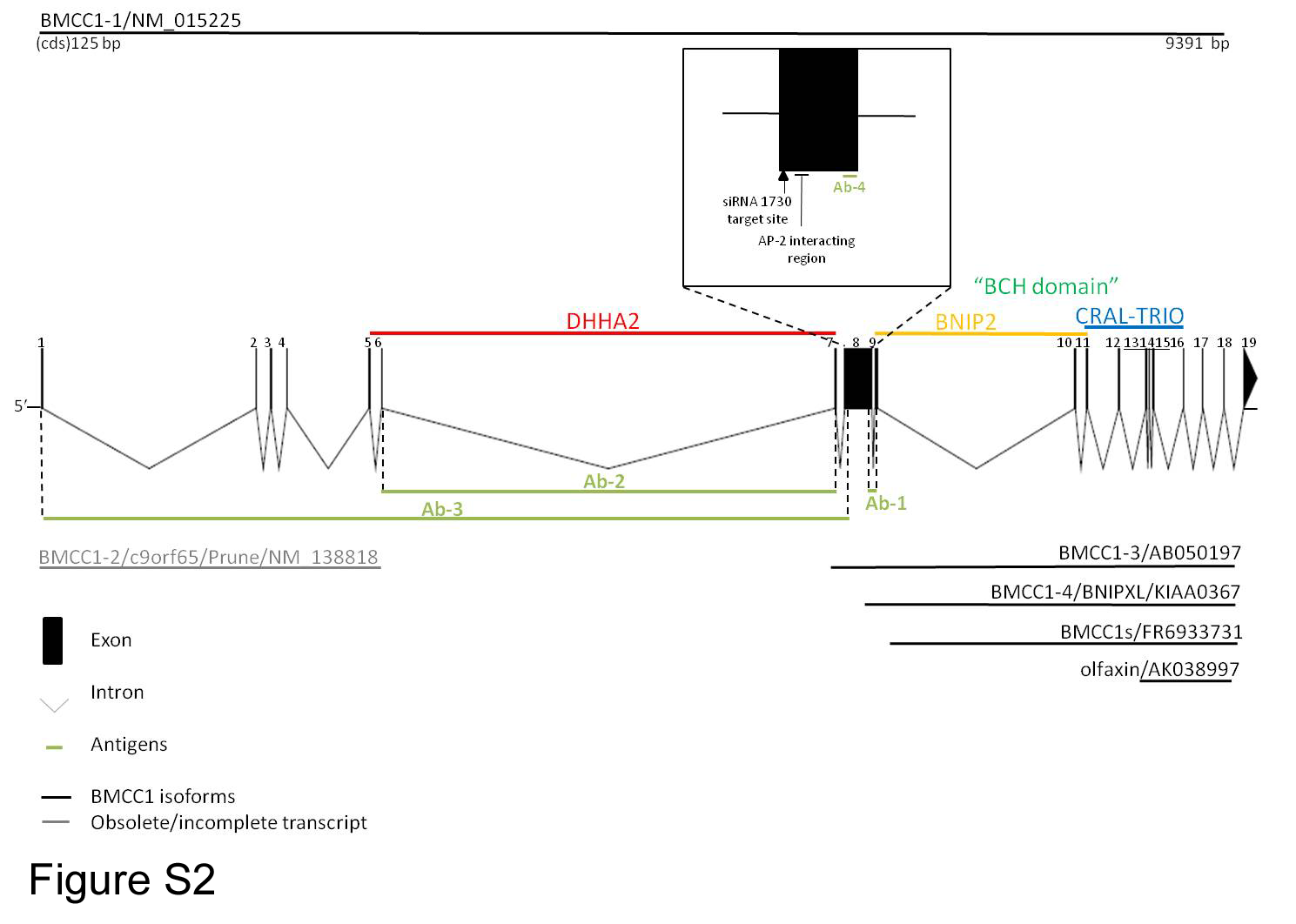

Supplement: Figure S2 — Schematic of BMCC1 gene structure and predicted protein domains. BMCC1 exon structure and protein domains were collated using the online tools ExDom, Exon-Intron Graphic Maker (wormweb.org) and Interpro: protein sequence analysis and classification. BMCC1 isoform 1 (BMCC1-1) RNA is depicted with reference to the smaller isoforms 2-4, BMCC1s and olfaxin (NCBI accession numbers are noted). The obsolete BMCC1-2 is included for illustrative purposes. The cDNA regions cloned for recombinant antigen expression and antibody production, as well as AP-2 pull-down (AP-2 interacting region) are indicated. The siRNA target region is also indicated. Note that all line depictions include intronic sequences and as such do not reflect translated peptide/protein size. (TIF) [file pone.0073880.s002.tif]

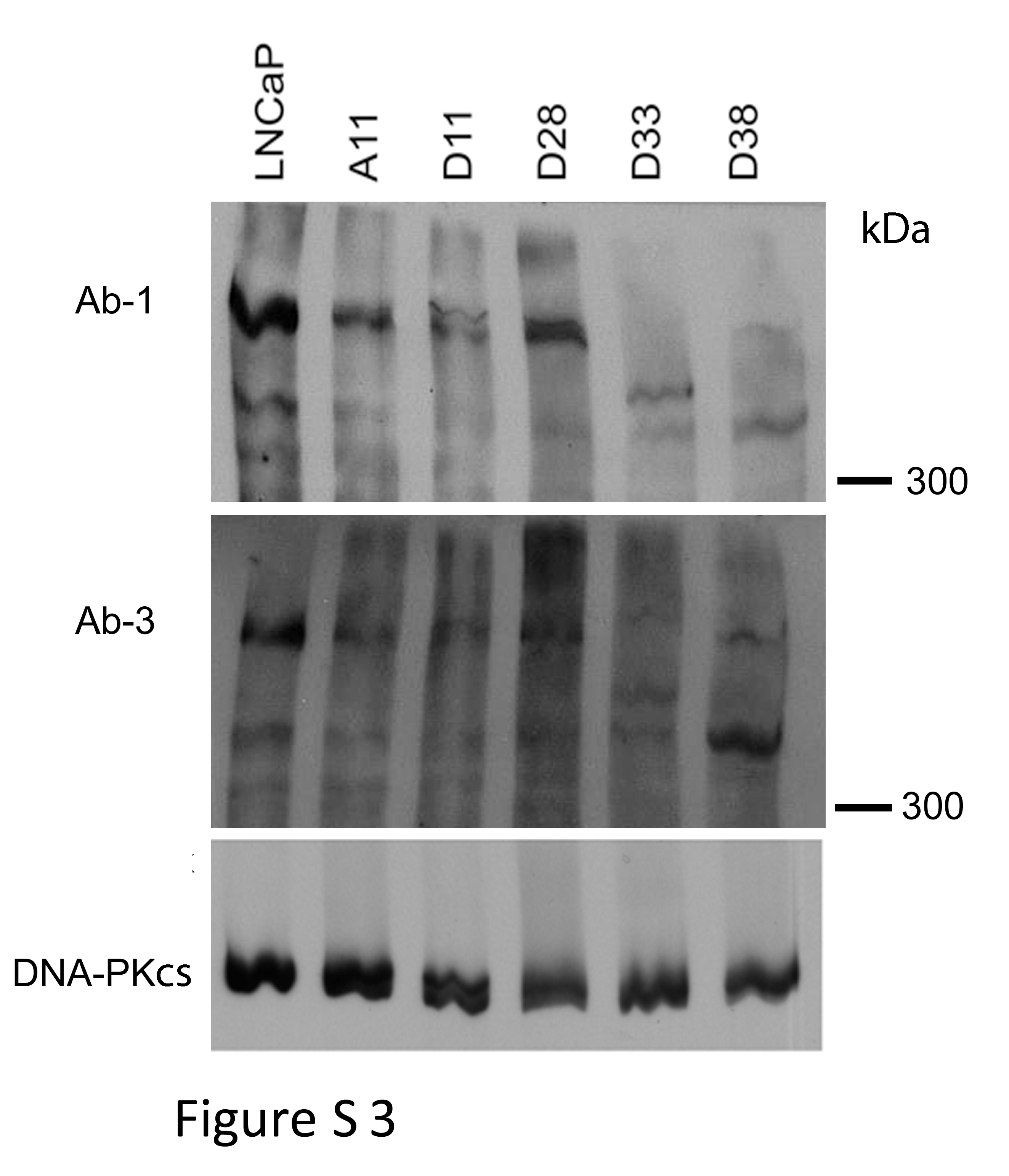

Supplement: Figure S3 — Expression of BMCC1 in LNCaP and melanoma cell lines. Total cell lysates were generated from the primary melanoma cell line A11, D11, D28, D33 and D38. 30 µg of total lysate from each cell line was subjected to SDS-PAGE and western blotting with rabbit anti-BMCC1 antibodies (Ab-1, Ab3). DNA-PKcs is shown as a loading control. (TIF) [file pone.0073880.s003.tif]

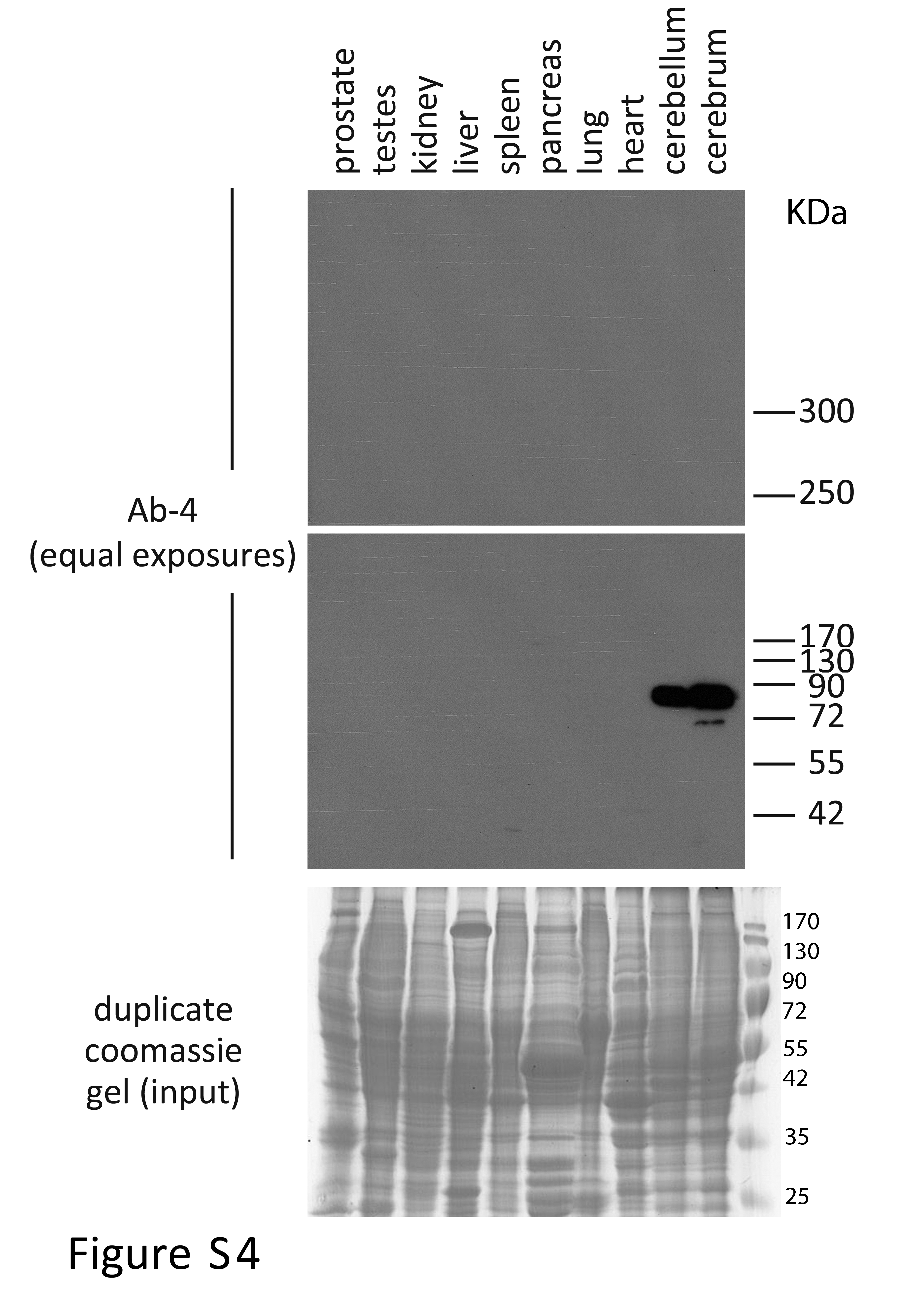

Supplement: Figure S4 — Tissue expression profiling. BMCC1 protein expression was examined in lysates from a healthy 12 week old male C57/B6 mouse. Tissues were harvested and lysed in MCLB without haemolysis, and 100 µg of clarified lysate from each tissue was resolved by SDS-PAGE on 5% or 5 to 15% gels. Proteins were detected by western blotting or Coomassie staining as indicated. (TIF) [file pone.0073880.s004.tif]

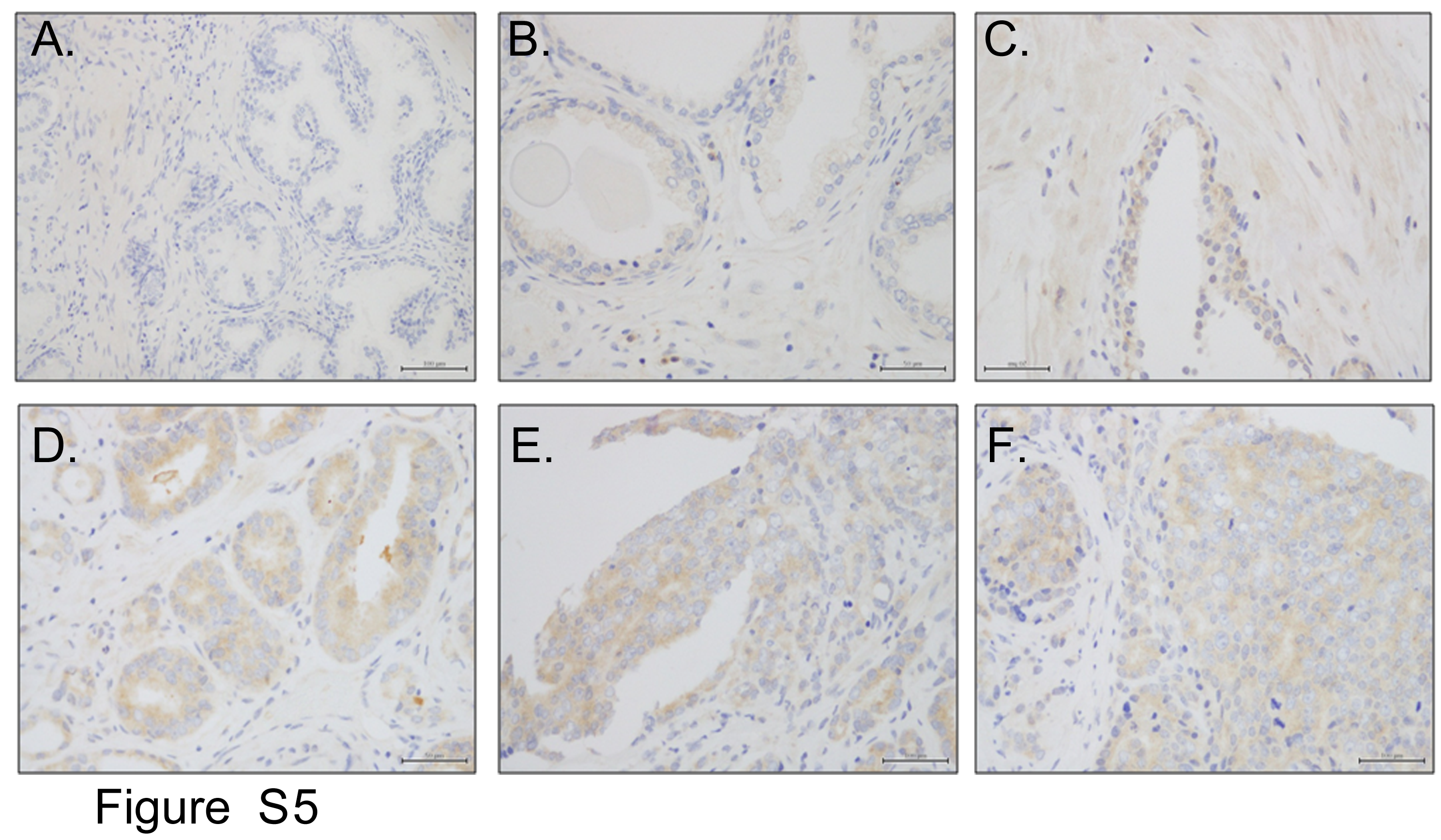

Supplement: Figure S5 — Immunohistological staining for BMCC1 in prostate cancer tissues. Benign prostatic hypertrophy (BP) (Panels A-C) and prostatic adenocarcinoma (PCa) (Panel D grade 3, Panel E grade 4, Panel F grade 5) tissue sections were incubated with buffer only (Panel A) or BMCC1 Ab-3 (Panels B–F) and detected using streptavidin-biotin-peroxidase immunostaining with diaminobenzidine. Size bar is 100µm (A, E, F) or 50µm (B, C, D). (TIF) [file pone.0073880.s005.tif]

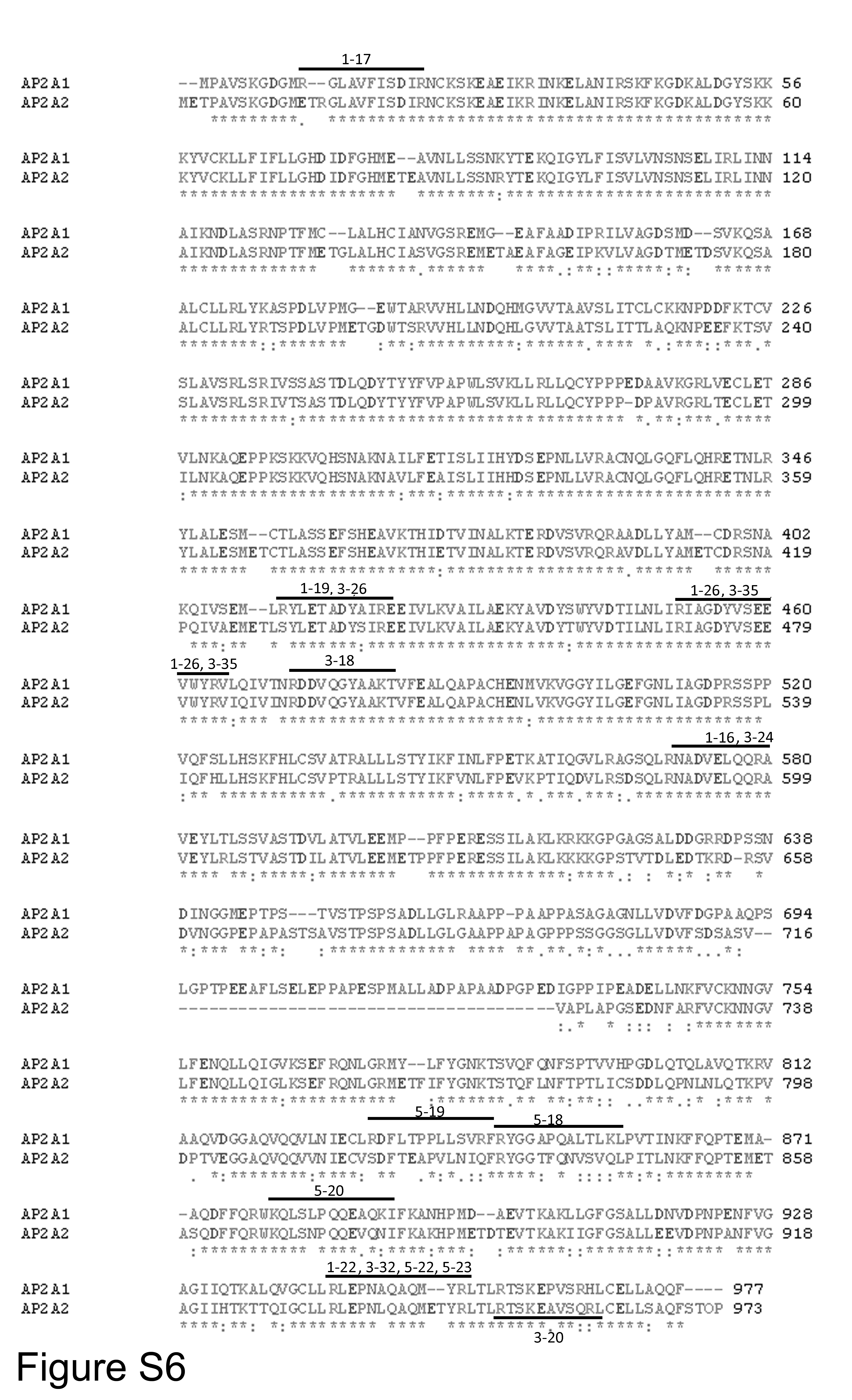

Supplement: Figure S6 — Alignment of AP-2A1 and AP-2A2. AP-2A1 and AP-2A2 protein sequences obtained from the NCBI were aligned using Clustal W (default settings). Peptides identified by MALDI MS/MS in our purification of BMCC1 interactors are indicated, identified by sample number- peptide number with summary statistics for each peptide in Supp Table 2. (TIF) [file pone.0073880.s006.tif]

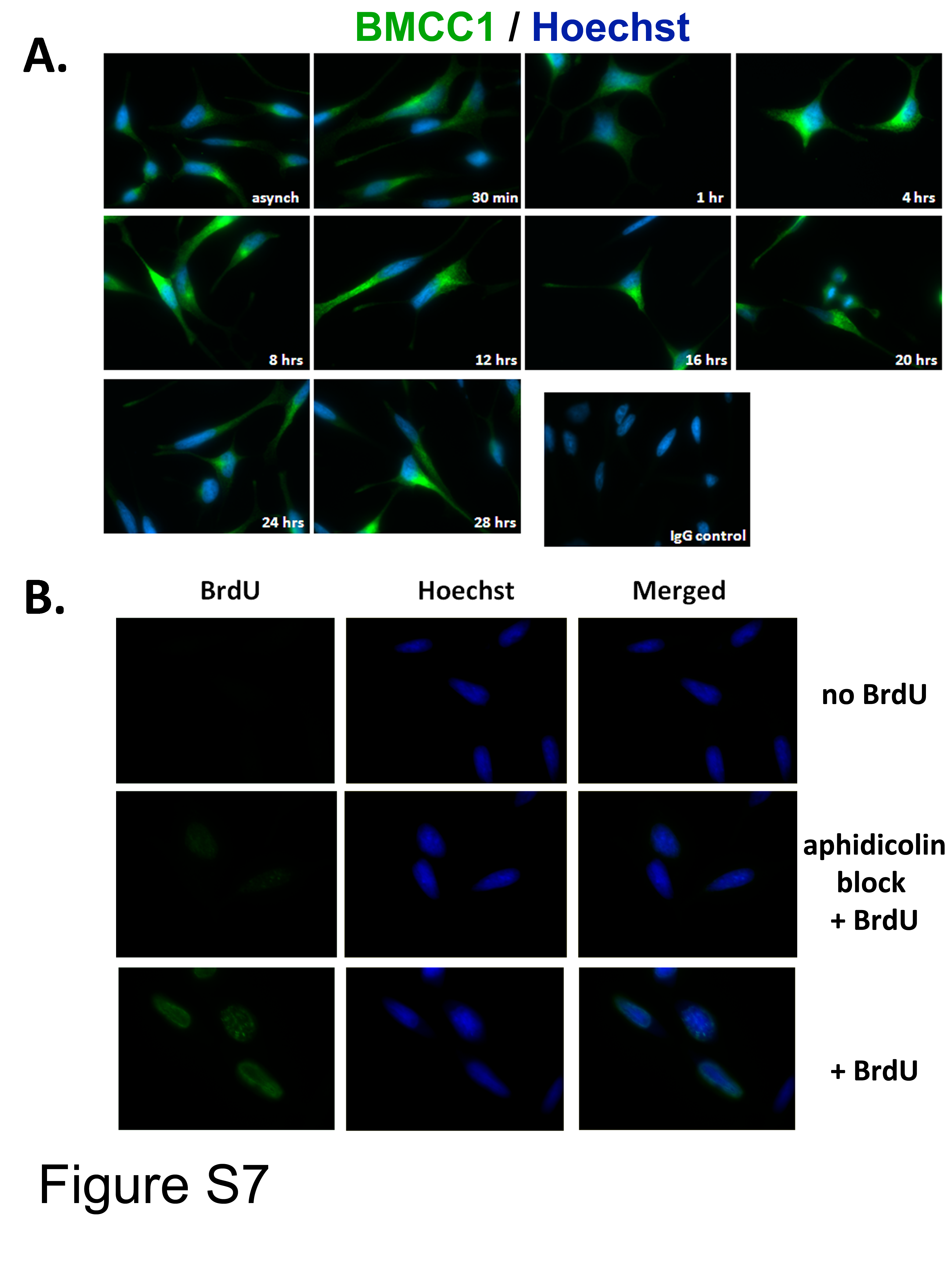

Supplement: Figure S7 — Expression of BMCC1 throughout the cell cycle. A. Sychronised LNCaP cells with a single aphidicolin block (5 µg/mL) for 36 h. Cells were released by washing in drug-free media and fixed at intervals after aphidicolin removal. Fixed coverslips were kept in PBS at 4 °C until all time points had been collected. Cells were then stained for BMCC1 (rabbit Ab-1 antibody) and detected with donkey anti-rabbit Alexafluor488. Cells were counterstained with Hoechst, mounted in moviol and analysed on a wide-field microscope (x63 objective). B. Inhibition of S-phase progression assessed by BrdU incorporation. Normally cycling or aphidicolin blocked cells were incubated with 100 µM BrdU for 2 h in growth media before fixation. BrdU incorporation was detected in fixed, alkaline denatured cells using rat anti-BrdU and donkey anti-rat Alexafluor 488. (TIF) [file pone.0073880.s007.tif]

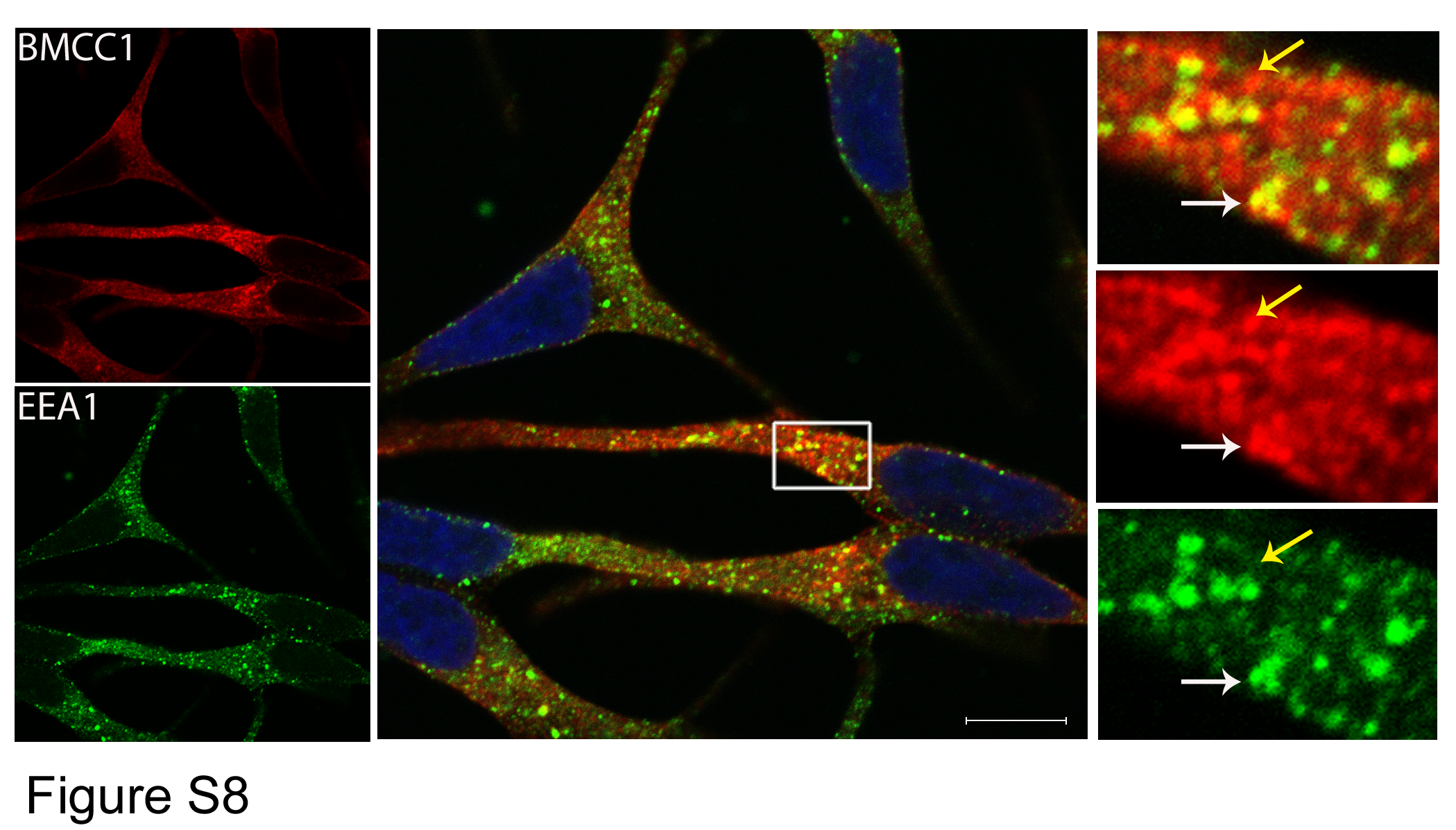

Supplement: Figure S8 — BMCC1 colocalisation and juxtaposition with early endosomal marker EEA1. LNCaP cells were stained for sheep anti- BMCC1 and mouse anti- EEA1, and detected with anti-sheep Alexafluor594 and anti mouse- Alexafluor 488. The white arrow highlights an area of clear co-localisation between the two proteins and the yellow arrow highlights an EEA1-negative BMCC1-positive vesicle. Scale bar is 10 µm. (TIF) [file pone.0073880.s008.tif]

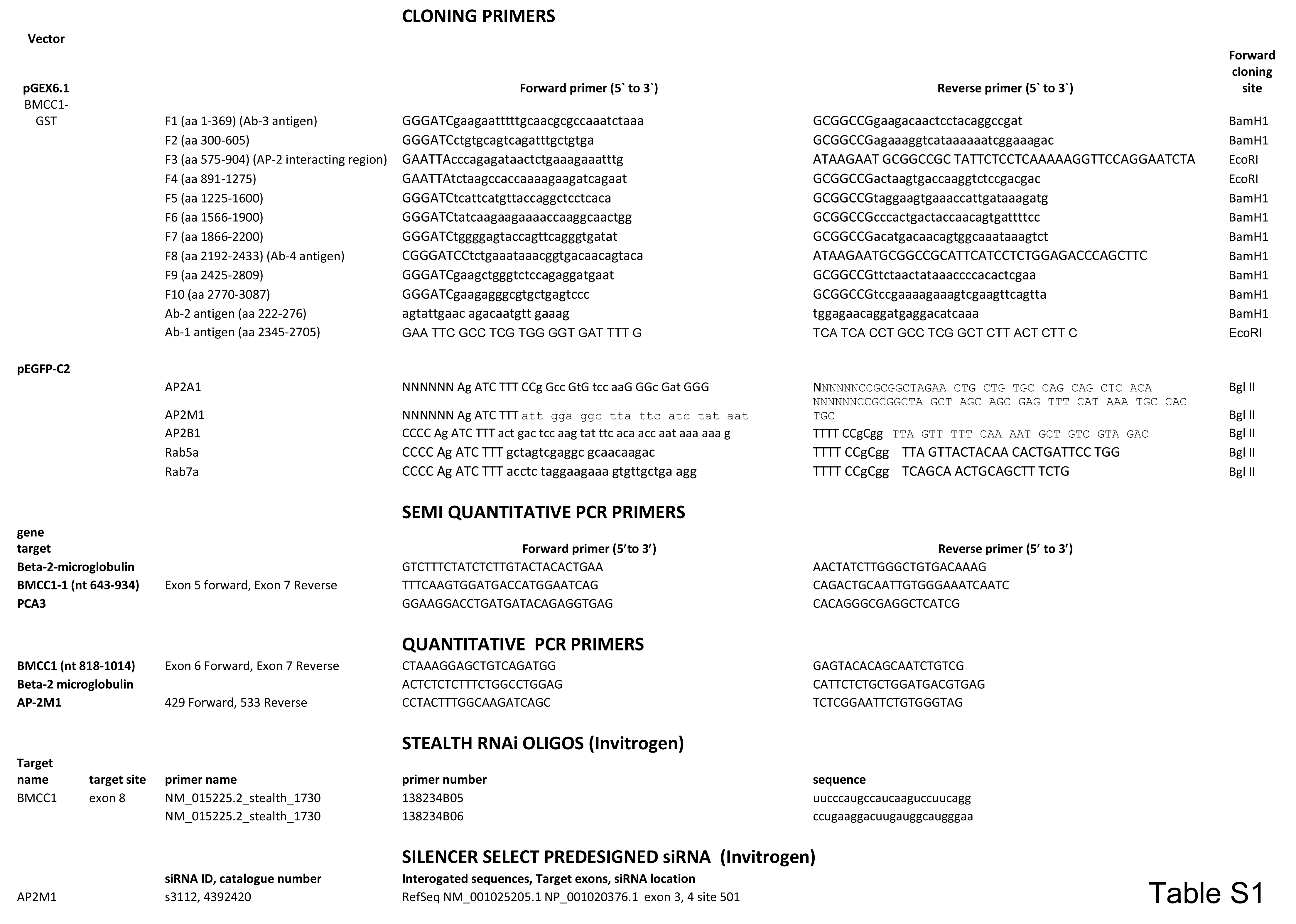

Supplement: Table S1 — Cloning, PCR and siRNA primer sequences. (TIF) [file pone.0073880.s009.tif]

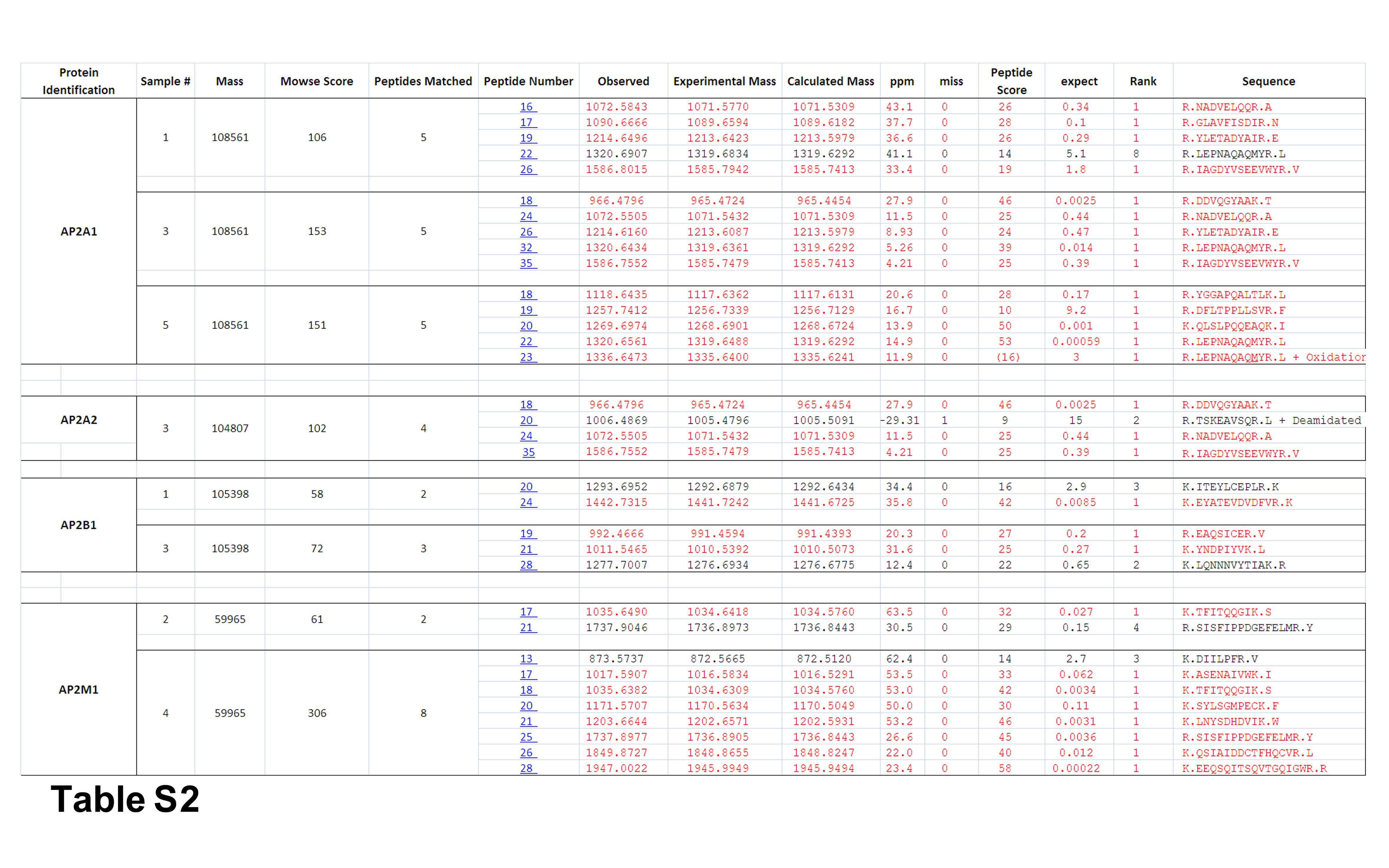

Supplement: Table S2 — MALDI TOF/TOF data summary- BMCC1 interactions. (TIF) [file pone.0073880.s010.tif]

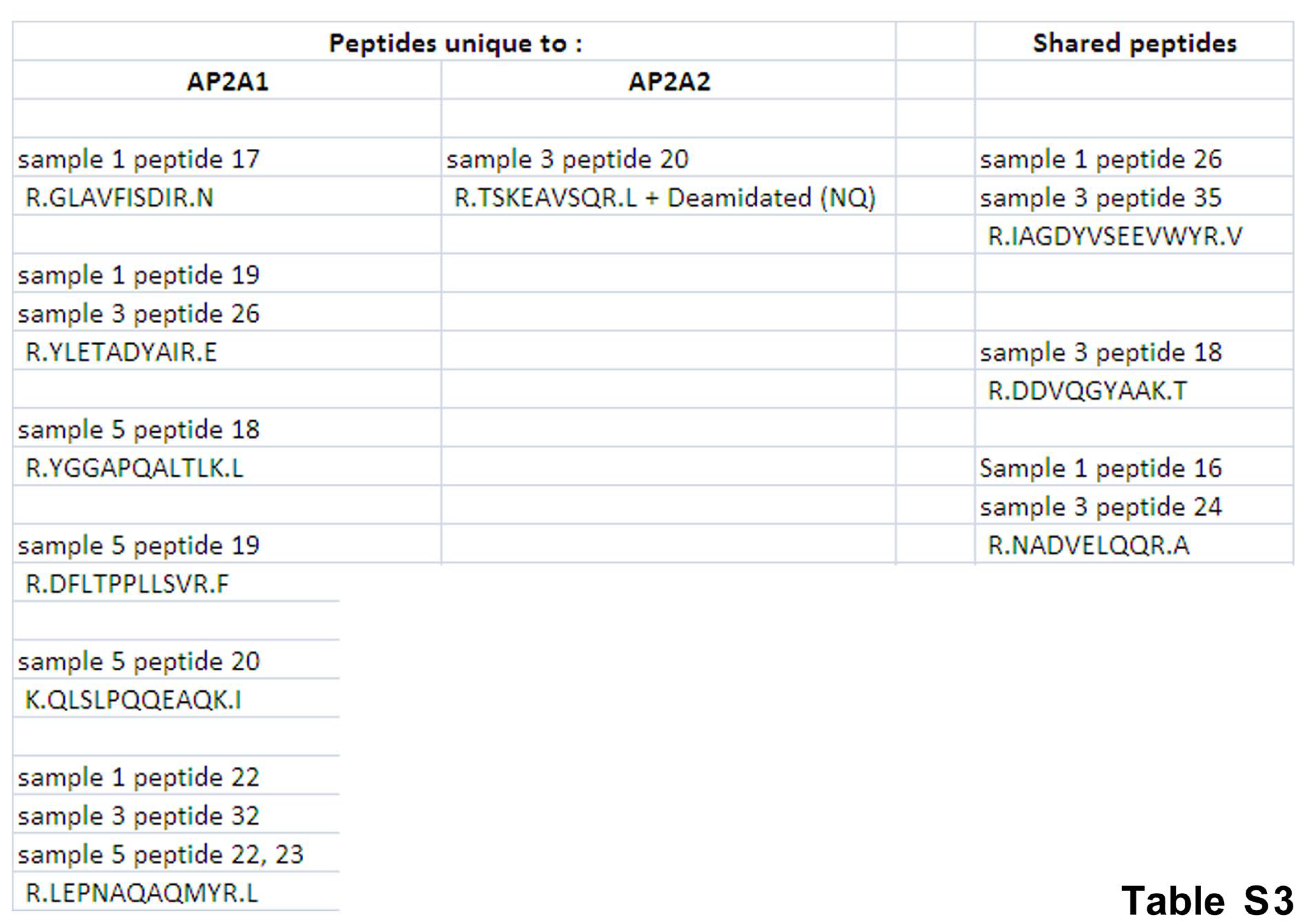

Supplement: Table S3 — Attribution of identified peptides between high-homology AP-2A1 and AP-2A2. (TIF) [file pone.0073880.s011.tif]

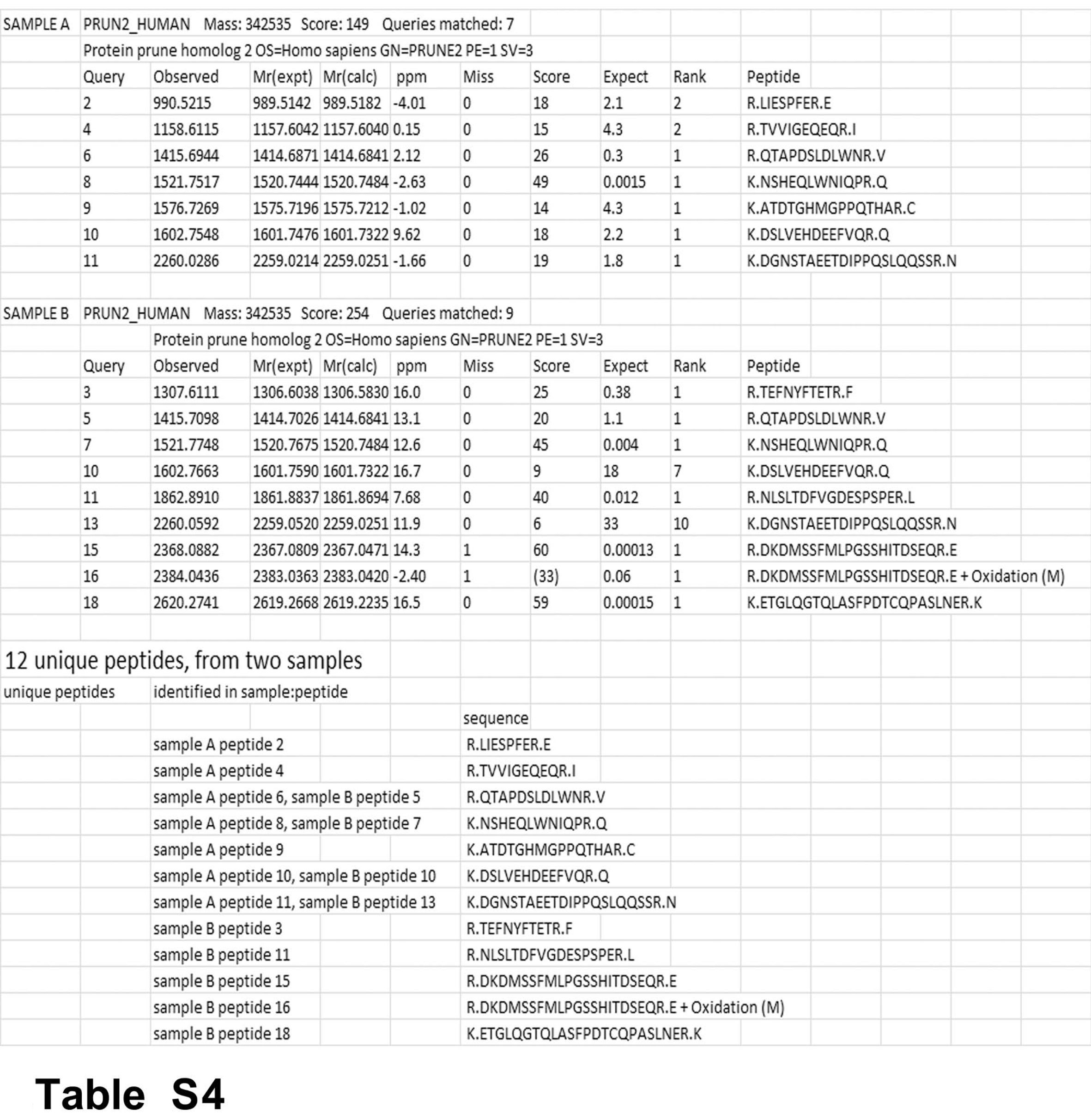

Supplement: Table S4 — MALDI TOF/TOF data summary- BMCC1 primary sequencing. (TIF) [file pone.0073880.s012.tif]
